# Amniogenesis occurs in two independent waves in primates

**DOI:** 10.1016/j.stem.2022.03.014

**Published:** 2022-05-05

**Authors:** Maria Rostovskaya, Simon Andrews, Wolf Reik, Peter J. Rugg-Gunn

**Affiliations:** 1 Epigenetics Programme, Babraham Institute, Cambridge CB22 3AT, UK; 2 Bioinformatics Group, Babraham Institute, Cambridge CB22 3AT, UK; 3 Altoslabs Cambridge Institute, Cambridge CB21 6GP, UK; 4 Wellcome Trust Sanger Institute, Hinxton, Cambridge CB10 1QR, UK; 5 Centre for Trophoblast Research, University of Cambridge, Cambridge CB2 3EG, UK; 6 Wellcome-MRC Stem Cell Institute, Cambridge CB2 0AW, UK

**Keywords:** human embryonic development, amnion, human pluripotent stem cells, pluripotency states transition, differentiation

## Abstract

In primates, the amnion emerges through cavitation of the epiblast during implantation, whereas in other species it does so later at gastrulation by the folding of the ectoderm. How the mechanisms of amniogenesis diversified during evolution remains unknown. Unexpectedly, single-cell analysis of primate embryos uncovered two transcriptionally and temporally distinct amniogenesis waves. To study this, we employed the naive-to-primed transition of human pluripotent stem cells (hPSCs) to model peri-implantation epiblast development. Partially primed hPSCs transiently gained the ability to differentiate into cavitating epithelium that transcriptionally and morphologically matched the early amnion, whereas fully primed hPSCs produced cells resembling the late amnion instead, thus recapitulating the two independent differentiation waves. The early wave follows a trophectoderm-like pathway and encompasses cavitation, whereas the late wave resembles an ectoderm-like route during gastrulation. The discovery of two independent waves explains how amniogenesis through cavitation could emerge during evolution via duplication of the pre-existing trophectoderm program.

## Highlights

• The amnion develops in two distinct waves of epiblast differentiation in primates
• The amniotic cavity is formed during the early wave via a trophectoderm-like route
• Late amniogenesis follows a nonneural ectoderm-like transcriptional program
• Differentiation of hPSC from different states models the two waves of amniogenesis

## Graphical abstract

**Figure:**
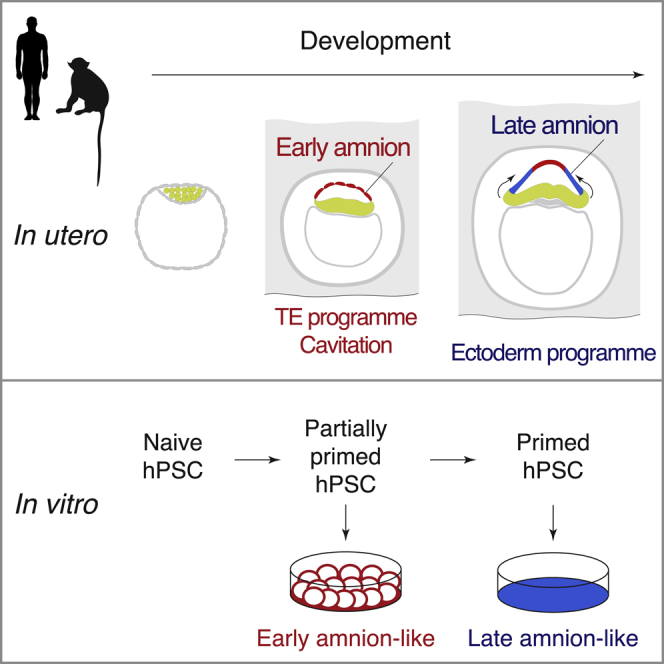

## Teaser

Lineage specification during human peri-implantation development remains poorly understood. Rostovskaya et al. discovered that in primate embryos the amnion is formed in two transcriptionally and temporally distinct waves of epiblast differentiation and established a human pluripotent stem cell-based model that closely recapitulates the timing and morphogenesis of both amniogenesis waves.

## Introduction

The amniotic membrane is an extraembryonic organ that forms a fluid-filled sac surrounding the embryo to provide mechanical protection and secrete hormones and cytokines (Mamede et al., 2012). Intriguingly, amnion specification follows strikingly dissimilar mechanisms in different species of mammals. In mice, rabbits, *Pteropodidae* bats, dogs, pigs, cows, and lower primates (lemurs, lorises, and galagos), the amniotic membrane is formed by the folding of embryonic tissues during or shortly after late gastrulation, whereby the amniotic folds extend and merge to form a closed sac (Pereira et al., 2011; Gopalakrishna and Karim, 1979; Carter and Mess, 2008; Dobreva et al., 2018; Perry, 1981; Greenstein and Foley, 1958; King, 1993). In other mammals, such as guinea pigs, hedgehogs, *Vespertilionidae* bats, and higher primates (monkeys and apes), the amnion emerges by delamination from pluripotent epiblast around the time of implantation, followed by epithelialization and cavitation, which produces the amniotic sac (Gopalakrishna and Karim, 1979; Carter and Mess, 2008; Enders et al., 1986; Luckett, 1975; Nakamura et al., 2016; Perry, 1981; King, 1993). Therefore, amnion formation occurs at discrete developmental points and by distinct morphogenetic events in different species and yet results in an anatomically and functionally similar organ. It remains unexplained how these dissimilar developmental mechanisms have evolved during phylogenesis.

Pluripotent stem cells are the *in vitro* counterparts of embryonic epiblast (Brook and Gardner, 1997; Boroviak et al., 2014; Nakamura et al., 2016). The developmental window of pluripotent epiblast extends from its emergence in the preimplantation blastocyst until lineage specification during gastrulation (Brons et al., 2007; Tesar et al., 2007; Osorno et al., 2012; Kojima et al., 2014). As such, two extreme states of pluripotency have been defined—naive and primed—corresponding to preimplantation and pregastrulation epiblast stages, respectively (Nichols and Smith, 2009). By using different culture conditions, human pluripotent stem cells (hPSCs) can be derived and propagated *in vitro* in these two distinct states (Thomson et al., 1998; Takashima et al., 2014; Theunissen et al., 2014; Guo et al., 2016), thus offering a unique opportunity to study embryonic peri-implantation events, including the segregation of cell lineages. Recent evidence suggests that naive hPSCs have the capacity to differentiate *in vitro* into other blastocyst lineages, including trophoblast (Castel et al., 2020; Dong et al., 2020; Cinkornpumin et al., 2020; Guo et al., 2021; Io et al., 2021; Kagawa et al., 2022; Yu et al., 2021; Liu et al., 2021) and primitive endoderm (PrE) (Linneberg-Agerholm et al., 2019). Primed hPSCs were reported to differentiate into cells resembling trophectoderm (TE), based on the expression of GATA2, GATA3, TFAP2A, TFAP2C, CDX2, and KRT7 markers (Xu et al., 2002; Amita et al., 2013), although these findings were at odds with the postimplantation identity of the primed hPSCs. This controversy has been resolved by recent evidence suggesting that the primed hPSCs produce amniotic epithelium rather than TE (Shao et al., 2017a, 2017b; Minn et al., 2020; Guo et al., 2021; Io et al., 2021) and that both lineages share the expression of the above markers. Paradoxically, primed hPSCs correspond to the epiblast at the onset of gastrulation, i.e., 14–16 days postfertilization (dpf), but the amniotic epithelium and the cavity emerge just after implantation (7 dpf) in human according to embryological data (Luckett, 1975). This inconsistency of timing of *in vitro* and *in vivo* amniogenesis remains unexplained. Therefore, an appropriate model of amnion specification is still missing, limiting our understanding of the mechanisms of lineage segregation and morphogenesis in the peri-implantation human embryo.

In this work, we analyzed published single-cell RNA-seq datasets from human and nonhuman primate embryos to elucidate developmental transitions during amnion specification. Unexpectedly, this revealed two transcriptionally distinct and temporally separated waves of amnion differentiation. Then we employed hPSCs corresponding to early and late postimplantation states and established an *in vitro* system that accurately recapitulates the timing, transcription profile, and morphogenetic events of each wave during amnion specification.

## Results

### The transcriptional blueprint of primate peri-implantation development

Several single-cell RNA-seq datasets describing the postimplantation development of primate embryos have recently become available, including *in vitro* cultured human pregastrulation embryos (Xiang et al., 2020), *in vitro* cultured cynomolgus monkey gastrulating embryos (Ma et al., 2019) and a human gastrulating embryo implanted *in utero* (Tyser et al., 2021). We aimed to integrate these three expression-profiling datasets to generate a blueprint of an extended peri-implantation period of primate embryo development. Human embryos from the Xiang et al. study were cultured until the onset of gastrulation, while cynomolgus embryos from the Ma et al. study were collected during gastrulation, and therefore we assumed that cynomolgus embryos were developmentally more advanced. The human gastrulating embryo implanted *in utero* had specified embryonic mesoderm and definitive endoderm; hence it was considered as the most advanced stage among these embryos.

**Figure 1:**
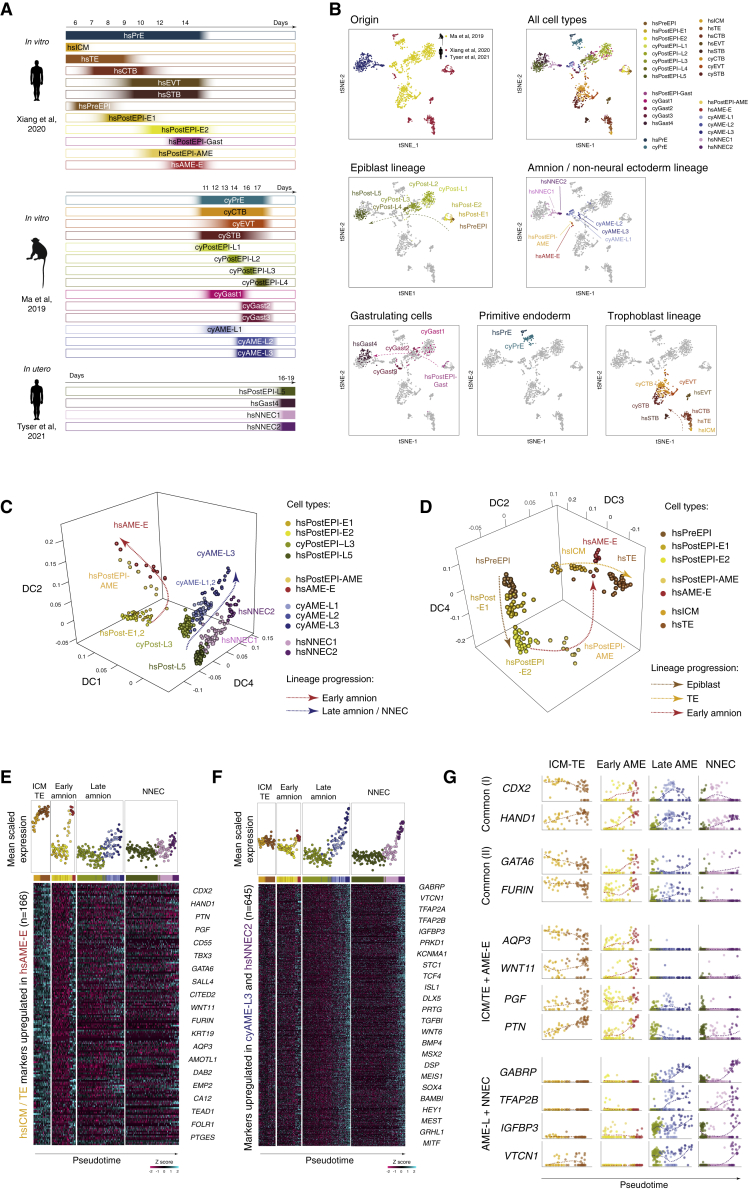
Two independent trajectories of amniogenesis in primate embryos (A) Cell types and their developmental stages within the integrated RNA-seq dataset. Note that developmental timing *in vitro* and *in utero* may differ. (B) tSNE of the integrated single-cell RNA-seq dataset of primate embryos. (C) Diffusion maps of amnion and non-neural ectoderm lineages in primate embryos. (D) Diffusion maps of early amnion and trophectoderm lineages in primate embryo. (E) Expression of the genes commonly upregulated in hsICM/hsTE and hsAME-E as compared with epiblast in embryo. (F) Expression of the genes commonly upregulated in cyAME-L3 and hsNNEC2 as compared with epiblast in embryo. (G) Expression of selected markers during lineages progression in embryo. See also Figures S1 and S2.

We curated cell annotations in these three individual datasets. For clarity, we harmonized the names of the cell subpopulations to a single style across the three datasets (Figure 1A). Initially, we and others (Chhabra and Warmflash, 2021) noticed that the original clustering of cell subpopulations of human pregastrulation embryos (Xiang et al., 2020) was affected by pseudogene expression, thus compromising the cell annotation. Therefore, we performed unsupervised clustering of this dataset considering only protein-coding genes (Figures S1A–S1C) and then reannotated based on the known markers (Figure S1D). We identified primitive endoderm (hsPrE), inner cell mass (hsICM), trophoblast lineage (hsTE, hsCTB, hsEVT, and hsSTB), epiblast lineage (hsPreEPI, hsPostEPI-E1 and -E2, and hsPostEPI-Gast), and two consecutive stages of amnion lineage (hsPostEPI-AME and hsAME-E).

Next, we refined the annotation of cynomolgus monkey single-cell RNA-seq data (Ma et al., 2019; Figure S1E) and identified TE lineage (cyCTB, cyEVT, and cySTB), four consecutive stages of epiblast lineage (cyPostEPI-L1, -L2, -L3, and -L4), primitive endoderm (cyPrE), gastrulating cells (cyGast-1, -2, and -3), and three subpopulations of the amnion lineage (cyAME-L1, -L2, and -L3).

Finally, we selected the cells defined as epiblast, primitive streak (PS) and nonneural ectoderm in a human *in utero* gastrulating embryo (Tyser et al, 2021), and grouped into four clusters (Figure S1F), comprising epiblast (hsPostEPI-L5), PS (hsGast4) and two stages of nonneural ectoderm (hsNNEC1 and hsNNEC2). Of note, the extraembryonic tissues of this embryo were manually removed prior to sample collection and therefore are unlikely to be present in the dataset. Therefore, we surmised that hsNNEC1/2 represent an early stage of surface ectoderm development rather than extraembryonic ectoderm, which is supported by expression of the diagnostic markers *TFAP2A*, *TFAP2B*, *MSX2*, *DLX5* (Figure S1F; Patthey and Gunhaga, 2014; Pieper et al., 2012; Streit, 2007; Qu et al., 2016).

The three single-cell RNA-seq datasets from human and cynomolgus monkey embryos were merged and normalized using Seurat (Figure 1B; Data S1 and S2). Dimensionality reduction analysis showed that cells of the same lineages from different datasets were co-localized in the tSNE (t-distributed stochastic neighbor embedding) plots, including trophoblast, epiblast, and PrE. Notably, the amnion and nonneural ectoderm cells also formed a cluster.

Analysis of diagnostic genes across the datasets confirmed our cell annotation and validated the strategy of integrating the data (Figure S1G). hsPrE and cyPrE cells were marked by *APOA1*, *PDGFRA*, *HNF4A*, and *FOXA2*. hsICM expressed *ESRRB*, and additionally shared a subset of markers with TE and/or epiblast (*TFCP2L1*, *DPPA3*, *POU5F1*, and *FOXH1*). Trophoblast lineage cells were marked by the expression of *TEAD3* and *AMOTL2*, in addition to markers shared with AME/NNEC (*GATA3*, *TFAP2A, CDX2,* and *HAND1*) lineages, consistent with previous reports (Nakamura et al., 2016; Ma et al., 2019). Genes characteristic for the AME/NNEC lineage included known markers *ISL1* and *BMP4* (Britton et al., 2019; Tchieu et al., 2017; Yang et al., 2021). Furthermore, other genes such as *GABRP*, *TFAP2B*, and *WNT6* were expressed at later stages of AME/NNEC development (cyAME-L3 and hsNNEC2).

Epiblast cells expressed the general pluripotency markers *POU5F1* and *DPPA4*, whereas other genes showed a dynamic pattern across the developmental progression of this lineage. Preimplantation epiblast (hsPreEPI)-specific genes included *FBP1* and *ARGFX* (Stirparo et al., 2018). A subset of genes including *TFCP2L1, KLF17, NODAL, FOXH1, NANOG, ETV4,* and *DPPA5* were downregulated after implantation in a distinct temporal order (Figure S1H). Postimplantation epiblast acquired expression of *SFRP2* and *SALL2*, followed by *FZD2* and *OTX2*. Overall, epiblast subpopulations of the three datasets formed a continuum of transcriptional changes, thus validating our annotation and assignment to developmental stages.

In summary, we have generated a comprehensive integrated single-cell RNA-sequencing dataset of human and nonhuman primate embryos. The epiblast progression in this dataset covers the period from the emergence of the ICM in the blastocyst until advanced gastrulation, thus offering an exceptional opportunity to investigate this period of development.

### Two independent trajectories of amnion specification in primate embryos

To investigate developmental transitions during amnion specification, we ordered the cells of the AME/NNEC cluster of the integrated RNA-seq dataset along their trajectories using diffusion map analysis (Figures 1C and S2A). Surprisingly, the trajectories of the early (from hsPostEPI-E to hsAME-E) and the late amnion (from cyPostEPI-L to cyAME-L3) diverged, suggesting the transcriptional independence and temporal separation of these lineages. The late amnion progression was closely aligned with the nonneural ectoderm trajectory.

Early amniogenesis in primates involves cavitation, which is also a hallmark of TE specification in preimplantation blastocysts. Therefore, we compared the developmental trajectories of TE and early amnion cells in embryos (Figures 1D, S2B, S2C, and S2D). Diffusion map analysis revealed their independent progression; however, hsAME-E converged with hsICM/hsTE cells in the DC2 component, thus reflecting their similarities.

Next, we analyzed the dynamics of gene expression in pseudotime during the progression of TE, early amnion, late amnion, and nonneural ectoderm lineages. First, we examined the expression of genes that distinguish hsICM/hsTE and hsAME-E (n = 166) from epiblast (Figures 1E and 1G; Data S1). Among this set, there were common genes upregulated in TE and amnion, including known factors, such as *CDX2* and *HAND1*, as well as not previously reported as amnion markers *GATA6* and *FURIN*. Importantly, most of the common hsTE/hsAME-E markers remained at low levels in the late amnion and nonneural ectoderm (Figures 1E and 1G). This included known hsTE-associated genes, such as *WNT11* and *PGF*, as well as a water channel *AQP3* that is critical for blastocyst cavitation (Watson and Barcroft, 2001). Second, we found a large number of genes (n = 645) commonly upregulated during the progression of late amnion and nonneural ectoderm lineages (Figures 1F and 1G; Data S1), including well-known factors involved in surface ectoderm development, such as *TFAP2B*, *WNT6*, *MITF*, *GRHL1*, and late amnion genes *ISL1*, *GABRP*, *HEY1*. Remarkably, the majority of these genes were not upregulated in hsAME-E.

In summary, our analysis of single cells from primate embryos unexpectedly revealed two distinct temporally separated transcriptional trajectories of amniogenesis, which therefore represent early and late amnion lineages. The early amnion recapitulated some transcriptional features of TE; in contrast, the late amnion shared similarity with nonneural ectoderm.

### hPSCs transiently gain the ability to form epithelial cavitating structures during the naive-to-primed transition

**Figure 2:**
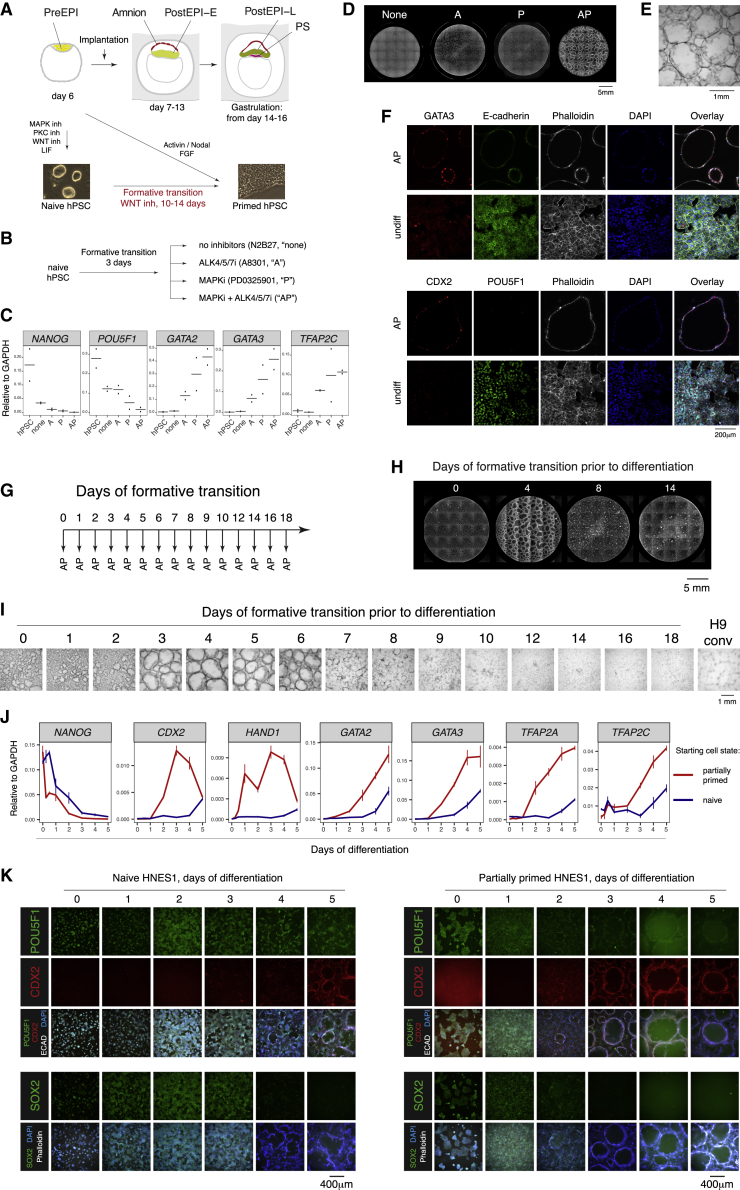
hPSCs transiently gain the ability to form epithelial cavitating structures during the naive-to-primed transition (A) A scheme of human peri-implantation development and correspondent hPSC states (PreEPI, preimplantation epiblast; PostEPI-E and PostEPI-L, early and late postimplantation epiblast, respectively; PS, primitive streak). (B) Experimental setup. Partially primed hPSCs were treated with either an inhibitor of ALK4/5/7, MAPK, or their combination. (C) qRT-PCR for markers after differentiation in indicated conditions; results of two independent experiments. (D) Stitched images of the cells in 24-well plates after differentiation in indicated conditions and staining with Phalloidin. (E) Bright-field image of 3D epithelial cavitating spheres obtained in AP condition. Note that the spheres remain attached to the surface of culture plates. (F) Immunofluorescence for GATA3 in combination with E-cadherin and CDX2 in combination with POU5F1, of partially primed hPSCs differentiated in AP condition (“AP”) and undifferentiated control (“undiff”). (G) Experimental setup. hPSCs on different days of the formative transition were differentiated in AP. (H) Stitched images of scanned 24-well plates showing hPSCs after different periods of the formative transition differentiated in AP (4′,6-diamidino-2-phenylindole (DAPI) staining). (I) Bright-field images of hPSCs after different periods of the formative transition and conventional H9 hPSCs differentiated in AP. (J) qRT-PCR for markers during the time course of AP treatment of naive and partially primed hPSCs. (K) Immunofluorescence of naive and partially primed hPSCs during the time course of differentiation in AP. See also Figures S3 and S4.

Primate peri-implantation embryos are difficult to access and to manipulate experimentally. Therefore, to further investigate amnion specification in human development, we sought to establish an *in vitro* model that is able to recapitulate amniogenesis. Naive and primed hPSCs are the counterparts of pre- and postimplantation epiblast, respectively; thus we employed an *in vitro* system for the naive-to-primed transition of hPSCs to access a peri-implantation epiblast-like state (Rostovskaya et al., 2019; Figure 2A). During this process, hPSCs accurately capture the dynamics of gene expression changes and upregulate members of the same signaling pathways as the epiblast cells during the progression from preimplantation to pregastrulation stage in cultured human embryos (Figures S3A–S3C; Xiang et al., 2020). Clonogenicity assays showed that most hPSCs irreversibly exited the naive state by day 3 of the formative transition (Figure S3D; Rostovskaya et al., 2019). Therefore, these partially primed hPSCs after 3 days of the transition were considered as closely matching the early peri-implantation epiblast and were used as a starting point for the amnion induction.

The naive-to-primed transition is guided by MAPK signaling in mouse PSCs and embryos (Kunath et al., 2007), whereas active TGF-β/NODAL signaling is required for hPSCs maintenance (Vallier et al., 2009; Osnato et al., 2021). Therefore, we tested whether the inhibition of these pathways using small-molecule inhibitors of MAPK (PD0325901) and ALK4/5/7 (A8301), respectively, can induce an amnion fate in partially primed hPSCs (Figure 2B). The repression of each of these pathways for 5 days resulted in the downregulation of the pluripotency genes *POU5F1* and *NANOG*, and the upregulation of the common TE/AME markers *GATA2*, *GATA3* and *TFAP2C* (Shao et al., 2017a). Furthermore, joint inhibition of MAPK and ALK4/5/7 (referred to as “AP” condition hereafter, A8301 + PD0325901) caused the largest and most consistent upregulation of these TE/AME genes (Figure 2C). Most strikingly, in AP conditions, numerous epithelial spheres spontaneously grew out of the monolayer while remaining attached to the surface of culture plates (Figures 2D and 2E; Videos S1 and S2). Immunofluorescence showed that the sphere-forming cells expressed GATA3, CDX2, and epithelial marker E-cadherin, and lacked the pluripotency factor POU5F1 (Figure 2F; Video S3).

Video S1. Time lapse imaging of partially primed HNES1 differentiated in AP, related to Figure 2 Timestamp indicates duration of the differentiation.

Video S2. Partially primed HNES1 differentiated in AP, bright field, z stack, related to Figure 2

Video S3. Immunofluorescence of epithelial spheres differentiated from the partially primed hPSC stained with: (left-hand side) CDX2 and POU5F1, (right-hand side) GATA3 and E-cadherin, both counterstained with Phalloidin and DAPI, related to Figure 2 Individual frames of these Z-stacks are shown in Figure 2F.

To identify a window of competence to produce these self-assembling cavitating spheres, we systematically probed the ability of hPSCs to respond to AP conditions during the formative transition (Figure 2G). The highest potential for the generation of the spheres in AP was observed between days 3 and 6 of the naive-to-primed transition (Figures 2H and 2I); thereafter, this ability rapidly declined and was not detectable beyond day 7-8 and for the conventional primed hPSCs.

We also observed some spheres in differentiated cultures obtained directly from naive hPSCs, but they were of a smaller size and at a lower number as compared with the cells derived from partially primed hPSCs. Naive hPSCs showed a consistent delay of about 24–48 h in the downregulation of pluripotency markers POU5F1, NANOG, and SOX2, and in the upregulation of the TE/AME genes CDX2, HAND1, GATA2, GATA3, TFAP2A, and TFAP2C, during AP induction as compared with partially primed hPSCs, validated by qRT-PCR (Figure 2J) and immunostaining (Figure 2K). A large proportion of naive hPSCs treated with AP still expressed POU5F1, indicating their resistance to differentiation. The delay and lower efficiency of generating the spheres were also confirmed by time-lapse microscopy (Video S4) of AP-induced naive hPSCs and partially primed hPSCs. Therefore, the exit from naive pluripotency is required for the competence to differentiate into the self-assembling epithelial spheres.

Video S4. Time lapse imaging of naïve and partially capacitated (for 3 days) HNES1 in AP differentiation conditions, related to Figure 2 Timestamp indicates duration of the differentiation.

Recent studies reported bone morphogenetic protein (BMP)-dependent differentiation of primed hPSCs to amniotic epithelium (Shao et al., 2017b; Guo et al., 2021; Io et al., 2021); therefore, we tested the effect of BMP in our differentiation system. The presence of BMP4 or an inhibitor of BMP receptor DM3189 did not interfere with the sphere formation and marker expression by the partially primed hPSCs in the AP condition (Figures S4A and S4B). The window of competence for formation of the cavitating structures during the naive-to-primed transition was not affected by exogenous BMP4 (Figure S4C). Remarkably, BMP inhibition significantly extended this window to days 3–8, with some spheres still emerging in cultures obtained from hPSCs even after 10 days of the transition. The decline in the ability to form spheres strongly correlated with the detection of neural markers PAX6 and SOX1 by flow cytometry and qRT-PCR upon BMP inhibition (Figures S4D and S4E). In contrast, beyond day 10 of the transition hPSCs upregulated markers of late amnion *GABRP* and *VTCN1* in AP or AP + BMP4 (Figure S4E). Therefore, primed hPSCs produce cells with characteristics of the late amnion upon joint inhibition of MAPK and NODAL in a BMP-dependent manner, in line with previous reports (Guo et al., 2021; Io et al., 2021), which we termed AME-L-like cells. In contrast, partially primed hPSCs differentiate to a distinct lineage through a novel BMP-independent process that has not been reported previously.

Taken together, hPSCs transiently gain the ability to form self-assembling epithelial structures during the naive-to-primed transition. The maximum capacity for doing so is observed in hPSCs after the irreversible exit from naive pluripotency and before the acquisition of primed features, thus closely matching the period of emergence of amnion during embryonic development. The differentiated cells spontaneously cavitate, express common TE/AME factors, and are devoid of late amnion markers, which together suggest an early amnion identity for these cells. We therefore termed them AME-E-like cells. As hPSCs reach the primed state, they switch this differentiation capacity to the ability to produce AME-L-like cells.

### Transcriptome profiling during AME-E induction of partially primed hPSCs

**Figure 3:**
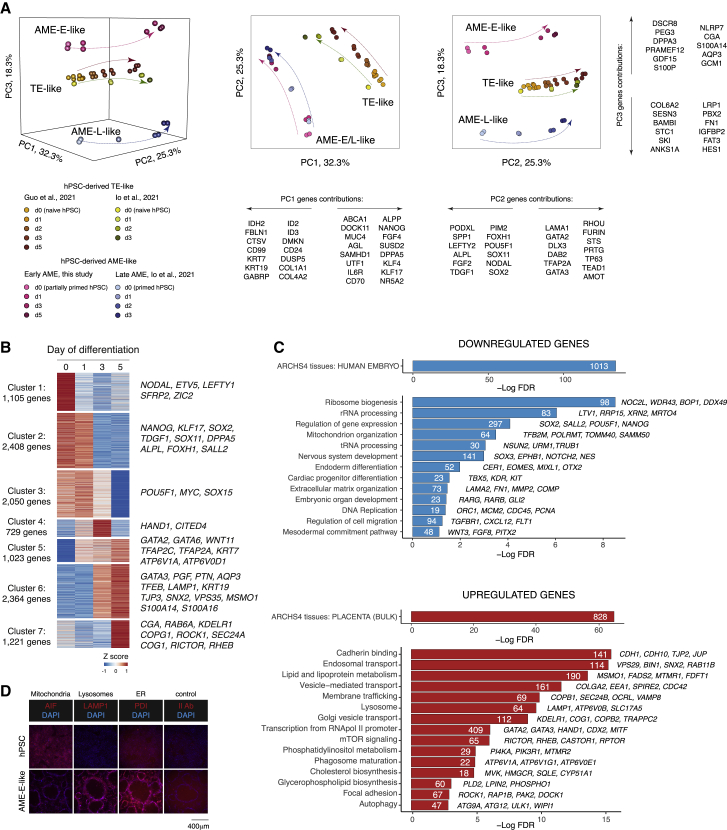
Characterization of AME-E-like cells transcriptome (A) PCA of transcriptomes during *in vitro* differentiation: naive hPSCs to TE-like, partially primed hPSCs to AME-E-like, and primed hPSCs to AME-L-like cells. (B) Heatmap showing clustering analysis of genes differentially expressed between any two time points of differentiation to AME-E-like cells. (C) Gene ontology analysis of differentially expressed genes between AME-E-like cells and hPSCs. (D) Immunofluorescence for organelles markers in AME-E-like cells and hPSCs.

Transcriptional changes during the differentiation of partially primed hPSCs to AME-E-like cells were characterized using bulk RNA sequencing. Because our analysis of primate embryos suggested that early amnion progression is independent of the late and involves an upregulation of hsTE features, we compared AME-E-like cells with the *in vitro* derived TE-like and AME-L-like cells differentiated from naive and primed hPSCs, respectively (Guo et al., 2021; Io et al., 2021), using principal component analysis (PCA) (Figure 3A). PC1 distinguished the TE-like and the combined AME-E/L-like cells, PC2 reflected the differentiation trajectory of all lineages showing their commonalities, whereas PC3 separated the three lineages. Analysis of gene loading to each PC identifies potential markers for these lineages, including *S100A14*, *S100P*, *AQP3*, *SAMHD1*, *GABRP*, and *STC1* (also investigated in more detail below). These results unambiguously show that AME-E-like cells produced by the partially primed hPSCs represent a unique lineage that is distinct from TE and late amnion.

Hierarchical clustering revealed 7 clusters of differentially expressed genes with various dynamics over 5 days of AME-E-like differentiation (Figure 3B; Data S3). Pluripotency factors, such as *TDGF1*, *POU5F1*, *NANOG*, and *DPPA5*, were among the most downregulated genes. Analysis of gene ontology terms (Figure 3C; Data S3) revealed that the downregulated genes encoded for proteins involved in somatic differentiation, RNA metabolism, mitochondrial functions, and DNA replication. The most upregulated genes included known common TE/AME markers (*GATA2*, *GATA3*, *TFAP2A, HAND1*, *KRT7*, and *KRT19*). Importantly, we observed an upregulation of the novel common hsTE/hsAME-E genes (*AQP3*, *CGA*, *PGF*, *PTN, GATA6,* and *WNT11*) identified in our analysis of embryos (Figures 1E and 1G). Upregulated genes included factors for adhesion, the mTOR (mammalian target of rapamycin) pathway, autophagy, lysosome activity, lipid and cholesterol biosynthesis, and membrane trafficking, consistent with the epithelialization, rapid increase of cell size, and membrane expansion events that are observed during cavitation. Immunofluorescence for organelle markers AIF (apoptosis-inducing factor), PDI (protein disulphide isomerase) and LAMP1 (lysosomal associated membrane protein 1) further confirmed the reorganization of intracellular structures, such as the reduction of mitochondrial content, expansion of the endomembrane system, and an increase in lysosomal activity (Figure 3D).

### Molecular signatures of trophectoderm and early and late amnion in primates

**Figure 4:**
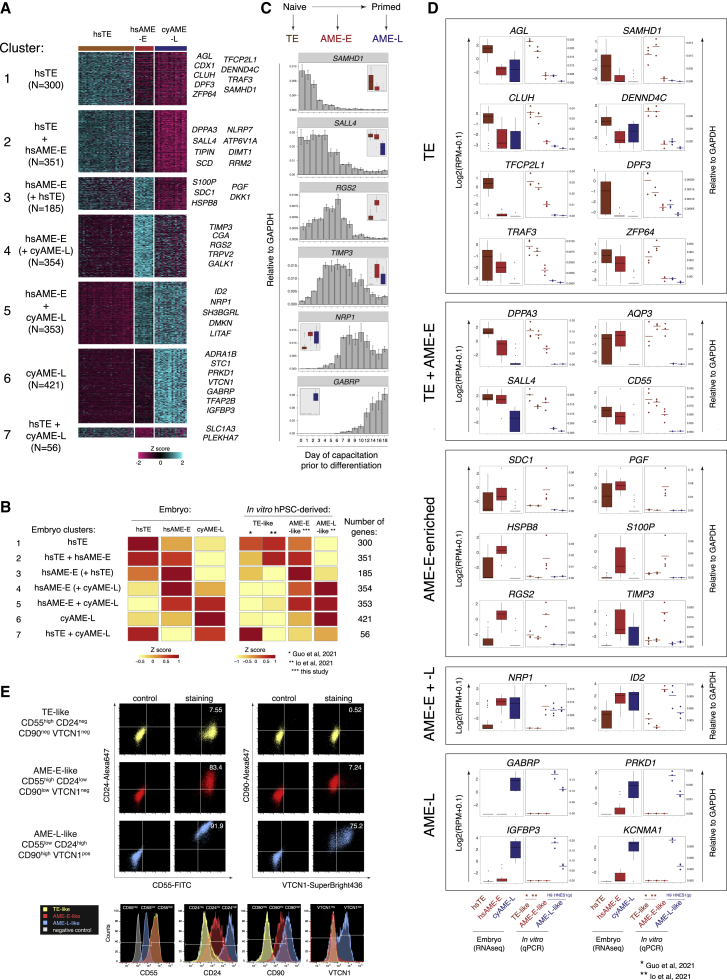
Molecular signatures of trophectoderm, early amnion, and late amnion in primates (A) Clustering analysis of all genes differentially expressed between any two cell types among hsTE, hsAME-E, and cyAME-L3. (B) Comparison of gene expression in embryo cell populations and their putative *in vitro* counterparts. The heatmaps show average expression levels of the gene clusters. (C) qRT-PCR of marker genes in cells differentiated in AP after various periods of the naive-to-primed transition. The insets show RNA-seq expression of the same markers in hsTE, hsAME-E, and cyAME-L3 of embryos. (D) Validation of markers. Boxplots show gene expression in embryos by RNA-seq. Scatter plots show *in vitro* differentiated cells by qRT-PCR. Each dot in the scatter plots indicates an independent differentiation experiment. (E) Flow cytometry of surface markers in the *in vitro* differentiated cells.

Our findings have provided evidence that there are two distinct pathways of amniogenesis in primate embryos, the early and the late, whereby the early pathway shared transcriptional similarity with the TE lineage. Next, we sought to identify the molecular signatures defining these three lineages, and for this we compared the transcriptomes of hsTE, hsAME-E, and cyAME-L3. Hierarchical clustering analysis of all genes differentially expressed between any of these cell populations revealed seven major clusters (Figure 4A; Data S4). Overall, hsTE, hsAME-E and cyAME-L3 showed expression patterns specific for each lineage; hsTE and cyAME-L3 had uniquely expressed genes (clusters 1 and 6, n = 300 and 421, respectively) and only a small fraction of common genes not detected in the hsAME-E (cluster 7, n = 56). In contrast, hsAME-E largely shared expression of markers with either of these lineages (clusters 2 and 5, n = 351 and 353 genes, respectively). Moreover, the genes enriched in hsAME-E were not exclusively expressed but were also detected in at least one of the alternative lineages (clusters 3 and 4, n = 185 and 354, respectively). Therefore, the early amnion lineage combines transcriptional features of both the TE and the late amnion.

**Figure 5:**
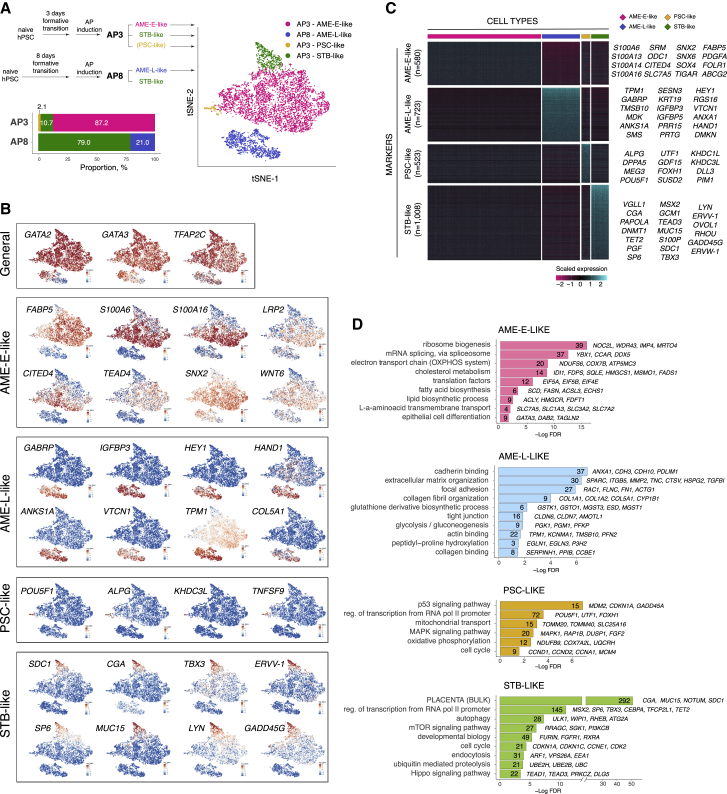
Characterization of amnion-like cell populations differentiated from partially primed and primed hPSCs at the single-cell level (A) tSNE of cell subpopulations differentiated from partially primed and primed hPSCs identified by 10× single-cell RNA-seq. (B) Selected markers of hPSC -derived subpopulations. (C) Heatmap showing expression of the markers of hPSC-derived subpopulations. (D) Representative gene ontology terms enriched among the markers of hPSC-derived subpopulations. See also Figure S5.

As the next step, we used the molecular signatures of hsTE, hsAME-E, and cyAME-L3 to validate the identity of their putative *in vitro* counterparts differentiated from naive, partially primed, and primed hPSCs, respectively, using the published RNA-seq datasets (Guo et al., 2021; Io et al., 2021) and our own data. Reassuringly, the embryo-expression pattern was largely reproduced by the *in vitro* differentiated TE-, AME-E- and AME-L-like cells (Figure 4B), thus confirming their identities. Additionally, we found that amnion cells differentiated from primed hPSCs using an independent protocol that includes BMP4 treatment (Zheng et al., 2019) expressed the late amnion markers rather than the early (Figure S5A). Therefore, hPSCs switch their differentiation competence from TE in the naive state, to the AME-E in the partially primed state and to the AME-L in the primed state. We validated a set of markers collectively defining these three lineages (*SAMHD1*, *SALL4*, *RGS2*, *TIMP3*, *NRP1*, and *GABRP*) in the cells treated with AP on each day of the naive-to-primed transition and found a pattern highly consistent with this conclusion (Figure 4C).

We verified a large set of the markers defining TE and early and late amnion in independent *in vitro* differentiation experiments using our own and published protocols (Guo et al., 2021; Io et al., 2021) by qRT-PCR (Figure 4D). These included TE (*AGL*, *SAMHD1*, *CLUH*, *DENND4C*, *TFCP2L1*, *DPF3*, *TRAF3*, and *ZFP64*); common TE/AME-E (*DPPA3*, *SALL4*, *CD55*, and *AQP3*); AME-E-enriched (*SDC1*, *PGF*, *S100P*, *HSPB8*, *RGS2*, and *TIMP3*); common AME-E/L (*NRP1* and *ID2*); and AME-L (*GABRP*, *PRKD1*, *IGFBP3*, and *KCNMA1*) markers. Additionally, we identified and validated a panel of surface molecules distinguishing the *in vitro* derived TE-like (CD55high, CD24neg, CD90neg, and VTCN1neg); AME-E-like (CD55high, CD24low, CD90low, and VTCN1neg); and AME-L-like (CD55low, CD24high, CD90high, VTCN1pos) cells (Figure 4E).

In summary, we have identified the molecular signatures defining TE, AME-E and AME-L in primate embryos and confirmed that our *in vitro* differentiation system faithfully recapitulates the formation of these extraembryonic tissues. We found a set of diagnostic markers defining TE, AME-E, and AME-L, including surface molecules that will facilitate further research and *in vitro* modeling of early human development.

### Single-cell transcriptional characterization of hPSC-derived amnion-like cells

We further focused on investigating the early and the late amnion lineages. We used 10X single-cell RNA-seq to characterize cell identities within the *in vitro* differentiated populations obtained by AP treatment of hPSCs that were primed for 3 days (“AP3”) or for 8 days (“AP8”) (Figure S5B). The 3D epithelial cavitating structures were readily formed in AP3, but not in AP8 cultures. Analysis of single-cell transcriptomes revealed four major categories of cells across these two cell populations (Figures 4A and S5C). First, AP3 was mostly (87.2%) composed of cells marked by the expression of *GATA3*, *S100A6*, *S100A16*, and *FABP5*; such cells were not found in AP8 (Figures 4B and S5D) and therefore were considered as AME-E-like. Second, AP8 cultures contained a unique cell population (20.9%) characterized by the expression of *GATA3*, *GABRP*, *HEY1*, and *IGFBP5*, which is consistent with an AME-L-like phenotype. Third, we detected a small (2.1%) subpopulation in AP3 cultures that expressed pluripotency genes *POU5F1*, *DPPA5*, and *NANOG*, but not the differentiation marker *GATA3*, indicating their failure to fully differentiate (“PSC-like cells” hereafter). Finally, subpopulations of cells with markers characteristic of syncytiotrophoblast (STB), such as *GATA3*, *PGF*, *PAPOLA*, *MSX2*, and *SP6* (Okae et al., 2018), were identified in both AP3 and AP8 cultures (“STB-like” hereafter).

To validate our assignment of cells to these four major categories across the samples, we integrated the AP3 and AP8 datasets (Figure S5E). Analysis using semisupervised category identification and assignment (SCINA) (Zhang et al., 2019) showed overall consistent results with the clustering of the separate datasets (Figures S5F, S5G, and S5H). The analysis of markers revealed that STB-like cells from AP3 and AP8 were not entirely equivalent (Figure S5I), but they shared major gene expression features, thus validating their assignment as STB-like cells.

In further analysis, we focused on the cells produced by partially primed hPSCs (AME-E-like, PSC-like, and STB-like), and AME-L-like that were produced by primed hPSCs. We identified markers specific for each category of cells and performed gene ontology analysis (Figures 4C and 4D; Data S5). The markers of AME-E-like cells were notably enriched for genes involved in phospholipid and cholesterol biosynthesis (*FDPS*, *FASN*, *MVD*, and *HMGCR*), transmembrane transport (*SLC7A5* and *SLC1A3*), and epithelial cell differentiation (*DAB2* and *TAGLN2*). AME-L markers included genes related to extracellular matrix and collagen biosynthesis (*COL1A1*, *COL5A1*, *GSTO1*, *EGLN1*, *SPARC,* and *SERPINH1*). PSC-like cells had higher expressions of genes involved in mitochondrial function and cell cycle, reflecting the transcriptional profile of undifferentiated hPSCs in our bulk RNA-seq. Finally, STB-like cells expressed numerous genes involved in placental development, as expected.

### hPSC-derived AME-E-like and AME-L-like cells transcriptionally resemble early and late amnion of primate embryos

**Figure 6:**
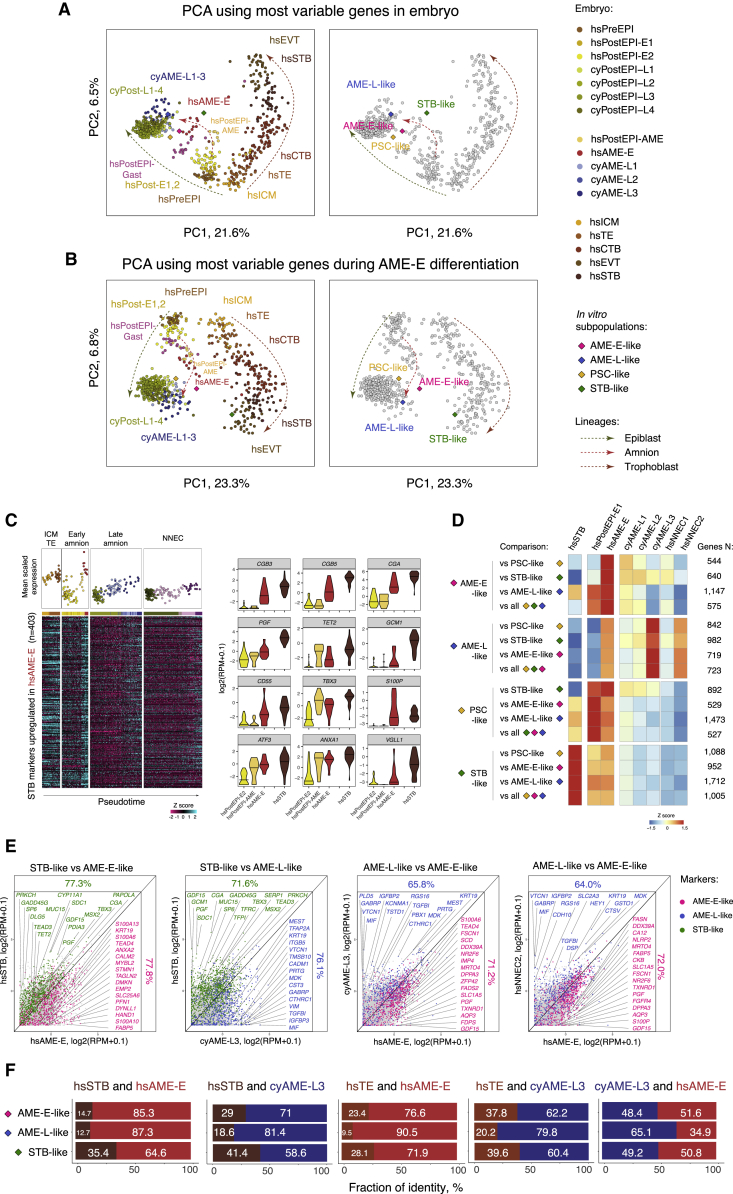
AME-E-like and AME-L-like cells transcriptionally resemble early and late amnion of primate embryos (A and B) PCA of the integrated primate embryo dataset and hPSC-derived subpopulations using the most variable genes of the embryo (A) and the most variable genes during *in vitro* differentiation (B). (C) Heatmap showing STB markers upregulated during the early amnion progression in embryos and violin plots of representative examples. (D) Heatmap showing average expression of the markers of hPSC-derived subpopulations in embryo. (E) Scatter plots showing pair-wise comparisons of the markers of hPSC-derived subpopulations, between the selected cell types in embryo. Percentage indicates a proportion of genes consistently upregulated in the respective lineage. (F) Fractions of identity of embryonic cell types in hPSC-derived subpopulations.

To precisely delineate the identity of hPSC-derived amnion-like cells, we performed PCA of the single-cell RNA-seq dataset from primate embryos with the pseudobulk expression of the four *in vitro* subsets that we identified by 10X sequencing. We used two gene sets for the PCA, either the most variable genes among the embryo cells or the genes variable during *in vitro* differentiation of hPSCs to AME-E (Figures 6A and 6B). In both cases, the trajectories of trophoblast, epiblast, and amnion lineages were resolved in the first two principal components. As expected, PSC-like cells were positioned on the pluripotent epiblast trajectory. STB-like cells were close to the amnion trajectory in the PCA plot obtained using variable genes of the embryo but clustered with the trophoblast lineage in the PCA performed using the variable genes during *in vitro* differentiation. Thus, STB-like cells likely represent a mixed identity resulted from the upregulation of STB-characteristic genes in amniotic epithelium. Most importantly, hPSC-derived AME-E-like cells clustered with hsAME-E cells, whereas AME-L-like cells grouped with cyAME-L3. This result was consistent in both analyses. Therefore, the PCA results strongly suggest that AME-E-like and AME-L-like cells belong to the amnion lineage corresponding to earlier and later developmental stages, respectively.

The upregulation of STB markers in our *in vitro* differentiation system was intriguing, especially because we did not observe substantial cell fusion associated with STB phenotype (Video S3) in differentiated cultures. We tested the dynamics of hsSTB markers during the early amnion lineage progression in the embryo and found that 403 genes out of 1,488 (27%) were upregulated (Figure 6C), including known STB-characteristic genes *CGB3*, *CGB5*, *CGA*, and *S100P*. Most of these genes were not induced in the late amnion or nonneural ectoderm lineages. Therefore, this partial upregulation of STB signatures is characteristic of the early amnion progression both *in vitro* and in the embryo.

Next, we performed differential gene expression analysis of our hPSC-derived subpopulations and identified markers characteristic for AME-E-like, AME-L-like, PSC-like, and STB-like cells in all pair-wise combinations, and additionally markers specific for each subpopulation as compared with the rest of the cells (Data S5). The expression of these markers was checked in selected cell types in embryos (Figures 6D and 6E). This analysis showed that the markers of AME-E-like cells had higher expression in hsAME-E. The genes characteristic for AME-L-like cells were enriched in cyAME-L3 cells; they were also moderately expressed in hsAME-E and hsNNEC2, indicating similarity of these lineages, in line with our previous findings (Figures 1 and 4). Finally, as expected, the markers of PSC-like and STB-like cells showed higher levels in hsPostEPI and hsSTB, respectively.

Finally, we assessed the identity of our four *in vitro* subpopulations using deconvolution analysis (DeconRNA-seq) (Gong and Szustakowski, 2013; Figure 6F). This method compares transcriptome of the cells of interest with other cell types and computes fractions of identity that reflect the relative similarity of the query to these cell types. We calculated the fractions of identity of hsAME-E in combinations with hsSTB or hsTE within our hPSC-derived subpopulations. The amnion identity prevailed in all the comparisons relative to the other cell types (64.6%–90.5%). The same was observed when the comparison was done with cyAME-L3 in combinations with the aforementioned cell types (58.6%–81.4%). Notably, the fraction of hsSTB was increased in STB-like cells as compared with the other subpopulations. The fraction of hsTE was higher in AME-E-like cells as compared with AME-L-like cells (23.4%–37.8% versus 9.5%–20.2%, respectively), consistent with our observations in the embryo. Finally, we probed the *in vitro* subpopulations against hsAME-E in combination with cyAME-L3. AME-L-like cells showed the largest fraction of cyAME-L3 identity (65.1%), confirming that AME-L-like cells are more developmentally advanced in amnion lineage progression than AME-E-like cells.

Taken together, our analysis showed that hPSC-derived AME-E-like and AME-L-like cells have amnion identity and represent earlier and later stages, respectively. Most importantly, our results suggest that AME-E-like cells can be produced only by the partially primed hPSCs, whereas AME-L-like cells only by the primed hPSCs, supporting our finding that the early and the late amnion cells are not two consecutive phases of differentiation, but independent, temporally separated lineages. Therefore, our *in vitro* system recapitulates the results of the developmental trajectories analysis using the transcriptome of primate embryos and provides the additional evidence for the two independent waves of amniogenesis in human.

**Figure 7:**
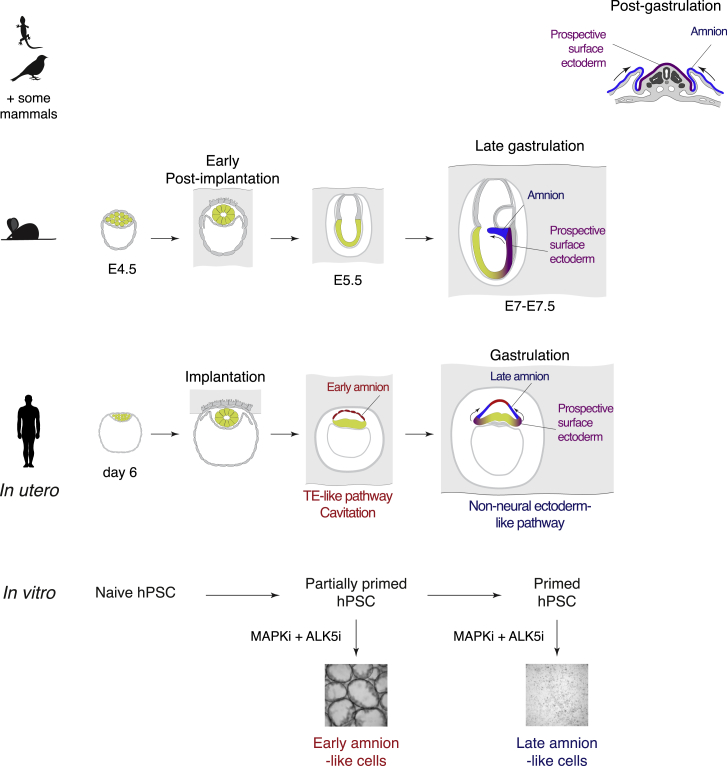
Amniogenesis occurs in two distinct waves in primate embryos The model of evolution of amniogenesis. Arrows indicate directions of morphogenetic movements.

In conclusion, we discovered that amniogenesis occurs in two independent waves in primate embryos (Figure 7). The early wave of differentiation occurs shortly after implantation and shows similarity to TE development, including transcriptional and morphogenetic features, such as cavitation. This is followed by a distinct, late wave of amniogenesis that begins from early gastrulation and relies on a nonneural ectoderm-like transcriptional program.

## Discussion

Formation of the amnion is a major adaptation of amniotes to terrestrial living, which enabled them to reproduce on land. While reptiles and birds share a significant similarity in the mechanisms of amniogenesis, there is a surprising variability in how the amnion and amniotic cavity emerge in developing mammalian embryos, and the reasons for this variability, as well as the mechanistic details of this process remain poorly understood. In our work, we discovered that amniogenesis occurs in two distinct waves in primate embryos. During the first wave, peri-implantation epiblast produces early amniotic epithelial cells that share multiple features with TE, including expression of the *AQP3* channel, potentially enabling them to initiate the cavity. The second wave ensues around the pregastrulation stage, when primed epiblast supplies additional cells to the amnion that exhibit transcriptional similarity to NNEC. The further fate of the early amniotic epithelium in embryos is not clear. We did not detect equivalent cells in gastrulating cynomolgus macaque embryos, either because of their low frequency or alternatively because they might represent a transient population which serves to initiate the cavity only then being eliminated afterward.

Recent reports have described the derivation of cells with characteristics of amnion from primed hPSCs (Shao et al., 2017a; Guo et al., 2021; Io et al., 2021). Nevertheless, the amnion emerges several days earlier than the epiblast reaches the primed stage in human embryos (Luckett, 1975) and this inconsistency was not explained. We established an *in vitro* system recapitulating both waves using hPSCs at different stages of the naive-to-primed transition. We found that partially primed hPSCs can differentiate to an epithelium that transcriptionally resembles early embryonic amnion and readily recapitulates the morphogenetic features of amnion formation, particularly cavitation. In contrast, primed hPSCs can differentiate to cells transcriptionally matching late amnion thus reproducing the second wave. These windows of competence to differentiate to the early and the late amnion-like cells during the naive-to-primed hPSCs transition *in vitro* accurately match the timing of the two waves in embryos according to histological and transcriptomic data (Luckett, 1975; Enders et al., 1986; Xiang et al., 2020; Ma et al., 2019). Therefore, our findings reconcile the seeming asynchrony between the ability of primed hPSCs to differentiate to amniotic epithelium and the timing of amniogenesis in the embryo. Interestingly, while AME-L-like cells grew as 2D monolayer in our work and several other reports (Guo et al., 2021; Io et al., 2021), Fu and co-workers observed formation of cavities when primed hPSCs were induced in a 3D gel-like culture system (Shao et al., 2017a, 2017b; Zheng et al., 2019). Therefore, it is possible that the amniotic epithelium of the late wave contributes to an additional expansion of the cavity after it has been initiated during the early wave.

The discovery of the early wave of amniogenesis particularly contributes to the understanding of the evolutionary divergence of amniogenesis (Figure 7). In reptiles and birds, the amnion is formed after gastrulation by folding (Patten, 1952; Blackburn and Flemming, 2009), and therefore this mechanism is possibly ancestral (van der Horst, 1949). In embryos with the folding type of amniogenesis, the amniotic epithelium lining the cavity forms a continuous layer with surface ectoderm and these lineages likely arise from common progenitors. In contrast, in multiple species of placental mammals, the amnion emerges around implantation through cavitation (Gopalakrishna and Karim, 1979; Carter and Mess, 2008; Enders et al., 1986; Luckett, 1975; Nakamura et al., 2016; Perry, 1981). Cross-species comparisons show that this mechanism likely evolved several times independently in different clades, which is difficult to explain. Here we propose that epiblast cells in these species “reuse” the existing transcriptional program of TE specification to initiate the amniotic cavity just after implantation, followed by the later wave of differentiation supplying more amnion cells around gastrulation through the conservative route resembling the folding mechanism. Repeated duplication of the pre-existing TE differentiation program for the early wave explains how the mechanisms of amniogenesis can be toggled between in evolution. Furthermore, reliance on the conserved surface ectoderm-like route of the late wave clarifies why amnion is anatomically and functionally similar in different species, regardless of the mechanism of its initiation.

An intriguing question is why amniogenesis needed adjustments during the evolution of placental mammals. It is possible that implantation and development within the uterine wall imposes topological constraints on morphogenetic movements, in particular the formation of amniotic folds. Different variations of embryogenesis might have evolved to allow for amniogenesis to proceed despite these constraints (Figure 7). It is conceivable that rodents adopted a cup-shaped cylindrical epiblast so that the proximal regions could merge to form the amnion following the folding-type mechanism. In contrast, primates (and some other species) evolved a precocious early wave of amniogenesis to form the cavity and to make space for the second wave of amnion differentiation. Interestingly, in pig and cow embryos, which implant after gastrulation and amniogenesis, the topology of amniotic folds strikingly resembles that of reptiles and birds (Patten, 1952; Greenstein and Foley, 1958), supporting our hypothesis. Additionally, the differences in mechanisms of amniogenesis could potentially influence the duration of epiblast progression. The transition of PreEPI to gastrulation takes about 10 days in human, but only 2 days in mouse embryos. This extended peri-implantation period might have evolved to accommodate the formation of the amniotic cavity in primates, while the development of rodents needed acceleration to reach the gastrulation stage and form the amnion for embryo protection. Indeed, the period between implantation and amnion formation is similar in mouse, human, and macaque embryos (Niu et al., 2019; Ma et al., 2019; Luckett, 1975).

The molecular basis of extraembryonic lineage specification is another pertinent question that our results touch upon. Our results and recent studies (Guo et al., 2021; Io et al., 2021) suggest that joint MAPK and NODAL inhibition induces TE, early amnion, and late amnion fates when applied to naive, partially primed, and primed hPSCs, respectively. Collectively, this demonstrates a continuous progression of differentiation competence of hPSCs to produce these three lineages during the naive-to-primed transition. Understanding the mechanisms of differentiation competence in hPSCs is an exciting prospect for future research.

### Limitations of the study

*In vitro* differentiation may not recapitulate all features of embryonic development.

Published single-cell RNA-seq datasets from human and macaque embryos were obtained using different sequencing protocols, which might have introduced bias in detection of some genes versus others.

We were only able to consider orthologous genes for integration of cynomolgus and human single-cell RNA-seq.

Only a limited number of human and cynomolgus macaque embryos were sequenced in Xiang et al. (2020) and Ma et al. (2019). We identified 11 cells of the early and 19 cells of the late amnion. More sequencing data from primate embryos are needed for more detailed analysis.

*In vitro* cultured human and cynomolgus embryos used for single-cell sequencing may differ from embryos *in utero*.

## STAR★Methods

### Key resources table

**Table:** 

REAGENT or RESOURCE	SOURCE	IDENTIFIER
**Antibodies**		

Polyclonal goat IgG anti-human SOX2	RnD Bio-Techne	Cat#AF2018; RRID:AB_355110
Polyclonal rabbit IgG anti-human CDX2	Cell Signaling Technology	Cat#3977S; RRID:AB_2077043
Monoclonal rabbit IgG anti-human GATA3	Abcam	Cat#ab199428 (EPR16651); RRID:AB_2819013
Polyclonal goat IgG anti-human E-cadherin	RnD Bio-Techne	Cat#AF748; RRID:AB_355568
Monoclonal mouse IgG2b anti-human POU5F1	Santa Cruz	Cat#sc-5279 (C-10); RRID:AB_628051
Monoclonal rabbit IgG anti-human Smad1	Cell Signaling Technology	Cat#6944 (D59D7); RRID:AB_10858882
Monoclonal rabbit IgG anti-human phospho-Smad1/5/9	Cell Signaling Technology	Cat#13820 (D5B10); RRID:AB_2493181
Mouse monoclonal IgG anti-human Hsp90	Abcam	Cat#ab13492 (AC88); RRID:AB_300396
Mouse monoclonal IgG anti-human PAX6 conjugated with PE	BD Biosciences	Cat#561552 (O18-1330); RRID:AB_10714781
Rabbit monoclonal IgG anti-human AIF	Cell Signaling Technology	Cat#5318 (D39D2); RRID:AB_10634755
Rabbit monoclonal IgG anti-human PDI	Cell Signaling Technology	Cat#3501 (C81H6); RRID:AB_2156433
Rabbit monoclonal IgG anti-human LAMP1	Cell Signaling Technology	Cat#9091 (D2D11); RRID:AB_2687579
Mouse monoclonal IgG anti-human CD55 conjugated with FITC	Biolegend	Cat#311305 (JS11); RRID:AB_314862
Mouse monoclonal IgG anti-human CD90 (THY1) conjugated with Alexa Fluor647	Biolegend	Cat#328115 (5E10); RRID:AB_893439
Mouse monoclonal IgG anti-human CD24 conjugated with APC	eBioscience, Invitrogen	Cat#17-0247-42 (SN3 A5-2H10); RRID:AB_10718833
Mouse monoclonal IgG anti-human VTCN1 (B7-H4) conjugated with SuperBright436	eBioscience, Invitrogen	Cat#62-5949-41 (H74); RRID:AB_2784834

**Chemicals, peptides, and recombinant proteins**		

MAPK inhibitor PD032590	Cambridge Stem Cell Institute facility	N/A
aPKC inhibitor Gö6983	Tocris Bio-Techne	Cat. 2285
Tankyrase inhibitor XAV939	Tocris Bio-Techne	Cat. 3748
Human leukemia inhibitory factor	Cambridge Stem Cell Institute facility	N/A
ROCK inhibitor Y-27632	Millipore	Cat. 688000
Activin receptor inhibitor A8301	Tocris Bio-Techne	Cat. 2939
BMP receptor inhibitor LDN193189 (DM3189)	Axon Medchem	Cat. 1509

**Critical commercial assays**		

Chromium Single Cell 3′ Library & Gel Bead Kit v3	10x Genomics	PN-1000075
Chromium Chip B Kit	10x Genomics	PN-1000073
Nextera XT kit	Illumina	FC-131-1024

**Deposited data**		

Raw and analysed data	This paper	GEO: GSE179309
Single-cell RNAseq of human *in vitro* cultured embryos	Xiang et al., 2020	GEO: GSE136447
Single-cell RNAseq of macaque *in vitro* cultured embryos	Ma et al., 2019	GEO: GSE130114
Single-cell RNAseq of human gastrula	Tyser et al, 2021	Array Express: E-MTAB-9388
Bulk RNAseq of the time course of naïve hPSC differentiation to TE-like cells	Guo et al., 2021	GEO: GSE166401
Bulk RNAseq of the time course of naïve hPSC differentiation to TE-like cells and primed hPSC differentiation to AME-L-like cells	Io et al., 2021	GEO: GSE144994
Single-cell RNAseq of *in vitro*-derived amnion-like epithelial cells	Zheng et al., 2019	GEO: GSE134571

**Experimental models: Cell lines**		

Human: HNES1	Guo et al., 2016	Naïve hPSC line HNES1
Human: cR-H9-EOS	Guo et al., 2017	Chemically reset hPSC line cR-H9-EOS
Human: H9 (WA09)	WiCell	hPSC line H9

**Oligonucleotides**		

Primers used in Figures 2, 4, and S4 and see Table S4	This paper	N/A

**Software and algorithms**		

RStudio	R Core Team, 2020	N/A
CellRanger v3.1.0	Zheng et al., 2017	N/A
Seurat V4.0.1	Hao et al., 2021	N/A
Ggplot	Wickham et al., 2019	N/A
Factoextra 1.0.7	Kassambara and Mundt, 2020	N/A
DESeq2	Love et al., 2014	N/A
Pheatmap	Kolde, 2019	N/A
SCINA	Zhang et al., 2019	N/A
DeconRNASeq	Gong and Szustakowski, 2013	N/A
Destiny	Angerer et al., 2016	N/A
ImageJ	Schneider et al., 2012	N/A
NIS-Elements	Nikon	N/A
Imaris	Oxford Instruments	N/A

### Resource availability

#### Lead contact

Further information and requests for resources and reagents should be directed to and will be fulfilled by the lead contact, Peter Rugg-Gunn (peter.rugg-gunn@babraham.ac.uk).

#### Materials availability

This study did not generate new unique reagents.

### Experimental model and subject details

#### Cell lines

The experiments were conducted using the embryo-derived HNES1 and the chemically reset cR-H9-EOS naïve hPSC lines (Guo et al., 2016, 2017), and conventional primed H9 hPSC (WA09, WiCell). The use of hPSC lines for these experiments has been approved by the UKSCB Steering Committee (SCSC11-58).

#### hPSC maintenance

Naïve hPSC were maintained on irradiated mouse embryonic fibroblasts (MEF) in PDLGX medium prepared as following: N2B27 supplemented with 1μM PD032590, 10ng/ml human LIF (both from Cambridge Stem Cell Institute facility), 2μM Gö6983 (Tocris Bio-Techne, Cat. 2285), and 2μM XAV939 (Tocris Bio-Techne, Cat. 3748), as described previously (Rostovskaya et al., 2019). N2B27 basal medium was prepared as following: Neurobasal (Cat. 21103049, ThermoFisher Scientific) and DMEM/F12 (Cat. 31331093, ThermoFisher Scientific) in the ratio 1:1; 0.5% N2 (Cat. 17202048, ThermoFisher Scientific), 1% B27 (Cat. 17504044, ThermoFisher Scientific), 2mM L-glutamine (Cat. 25030024, ThermoFisher Scientific), 100μM 2-mercaptoethanol (Cat. M7522, Sigma-Aldrich). Geltrex (A1413302, ThermoFisher Scientific) was added at a concentration 0.5μl/ml to the culture medium during re-plating. Naïve hPSC were passaged using TrypLE Express (Cat. 12604021, ThermoFisher Scientific) as single cells. 10μM ROCK inhibitor (Y-27632, Cat. 688000, Millipore) was added for 24 hours after passaging.

Conventional primed H9 hPSC (WA09, WiCell) were cultured in mTeSR-E8 media (Cat. 05990, STEMCELL Technologies) (Chen et al., 2011) on Geltrex pre-coated plates and passaged using 0.5mM EDTA in PBS. All cells were cultured in a humidified incubator with 5% O_2_ and 5% CO_2_ at 37°C.

#### Formative transition

The formative transition (capacitation) was performed as described previously (Rostovskaya et al., 2019). Prior to the formative transition, naïve hPSC were passaged once feeder-free to reduce the number of fibroblasts in the culture. For this, naïve hPSC cultured on MEF were dissociated with TrypLE Express, plated in the medium for naïve hPSC maintenance supplemented with 10μM ROCK inhibitor to non-coated tissue culture grade plates, and then Geltrex was added directly to the cells at a final concentration 1μl/cm^2^. For the formative transition, naïve hPSC were dissociated with TrypLE Express and plated onto Geltrex-coated tissue culture plates at a seeding density of 1.6x10^4^/cm^2^ in the media for naïve hPSC maintenance supplemented with 10μM ROCK inhibitor. After 48 hours, the cells were washed with DMEM/F12 supplemented with 0.1% BSA and the medium for capacitation was applied. Capacitation was performed using N2B27 supplemented with 2μM XAV939. The medium was refreshed every 1-2 days. The cells were passaged at confluency by dissociation using TrypLE Express and plating to Geltrex pre-coated dishes with a dilution 1:2; 10μM ROCK inhibitor was added for 24 hours after dissociation.

#### *In vitro* differentiation of hPSC

For differentiation to AME-E-like cells, partially primed hPSC were dissociated to single cells using TrypLE Express and counted. The cells were plated to Geltrex-coated tissue culture plates at a seeding density of 1x10^5^/cm^2^ in differentiation medium with 10μM ROCK inhibitor, and further cultured for 5 days. Differentiation medium was prepared as following: N2B27 basal medium, 1μM PD0325901 and 1μM A8301 (Cat. 2939, Tocris Bio-Techne). 100nM LDN193189 (alternative name DM3189, Cat. 1509, Axon Medchem) or 20ng/ml BMP4 (Miltenyi Biotec) were optionally added to the medium. The medium was changed daily. When the spheres appeared, the medium was refreshed by careful exchange of half volume of the medium, and the volume of the medium per well was increased.

Differentiation to TE-like cells was performed according to Guo et al. (2021) and Io et al. (2021). For differentiation to AME-L-like cells, primed hPSC were dissociated to single cells using TrypLE Express and counted. The cells were plated to Geltrex-coated tissue culture plates at a seeding density of 1x10^5^/cm^2^ in differentiation medium with 10μM ROCK inhibitor, and further cultured for 5 days. Differentiation medium was prepared as following: N2B27 basal medium, 1μM PD0325901, 1μM A8301 (Cat. 2939, Tocris Bio-Techne) and 20ng/ml BMP4 (Miltenyi Biotec). The medium was changed daily.

### Method details

#### qRT-PCR

Total RNA was extracted using RNeasy Mini Kit (74104, Qiagen) and 500ng was used for reverse transcription using RevertAid First Strand cDNA Synthesis kit (ThermoFisher Scientific). Quantitative PCR was performed with Brilliant III Ultra-Fast SYBR Green qRT-PCR Master Mix (Agilent). Primer sequences are listed in key resources table.

#### Western blot

Whole cell extracts were prepared by resuspending in a buffer containing (20mM Hepes pH8.0, 350mM NaCl, 10% glycerol, 0.1% Tween-20, 2mM EDTA, 1X Protease Inhibitor Cocktail (Roche) and 2% Phosphatase Inhibitor Cocktail (Abcam)) followed by 3 rounds of freezing in liquid nitrogen and thawing. 20μg of proteins were resolved in 4-12% polyacrylamide gel (NuPAGE, Invitrogen) and transferred to nitrocellulose membrane using iBlot Gel Transfer system (Invitrogen). Blocking was done overnight in 5% milk in Tris-buffered saline with 0.1% Tween-20 at +4°C. The membranes were incubated with primary antibodies diluted in the blocking solution for 1 hour at room temperature, washed Tris-buffered saline with 0.1% Tween-20, and incubated with secondary horseradish peroxidase-conjugated goat anti-rabbit or anti-mouse antibodies in the same conditions. The primary antibodies were used with the following dilutions: monoclonal rabbit IgG anti-human Smad1 (#6944, Cell Signaling Technology) 1:1000; monoclonal rabbit IgG anti-human phospho-Smad1/5/9 (#13820, Cell Signaling Technology) 1:1000; monoclonal mouse IgG anti-human Hsp90 (ab13492, Abcam) 1:1000. The secondary antibodies were diluted 1:5000.

#### Flow cytometry

Cells were dissociated using 0.05% Trypsin and 0.02% EDTA solution, and washed using PBS with 2% FCS. For surface marker staining, cells were incubated with directly conjugated antibodies diluted 1:50 in PBS with 2% FCS for 30min at +4°C, followed by washing and resuspending in PBS. The following antibodies against cell surface proteins were used: anti-CD55-FITC (Cat#311305, Biolegend), anti-CD90-AlexaFluor647 (Cat#328115, Biolegend), anti-CD24-APC (Cat#17-0247-42, eBioscience, Invitrogen), anti-VTCN1-SuperBright436 (Cat#62-5949-41, eBioscience, Invitrogen).

For intracellular markers staining, the cells were incubated in Fixation Buffer (00-8222-49, ThermoFisher Scientific) for 30min at +4°C, washed with Permeabilization Buffer (00-8333-56, ThermoFisher Scientific), and stained with anti-PAX6 (Cat#561552, BD Biosciences) antibody diluted 1:100 with Permeabilization Buffer and 5% donkey serum for 1 hour at +4°C. Detection was done using a BD Fortessa instrument (BD Biosciences) with analysis using FlowJo software.

#### Immunofluorescence

The cells were plated to 8-well chambered microslides (Cat. 80826, IBIDI) or standard tissue culture 24-well plates. For staining of AME-E-like cells, all washing steps were performed by careful exchange of half of the liquid volume to preserve the 3D structures. The cells were fixed with 4% formaldehyde in PBS for 15min followed by three washes in PBS. Cell permeabilization was done with 0.5% Triton X-100 in PBS for 10min followed by three washes in PBS. The cells were incubated with a blocking solution containing 3% BSA and 0.1% Tween-20 in PBS for 30min. All steps above were performed at room temperature. Incubation with primary antibodies diluted in the blocking solution was done overnight at +4°C. The following primary antibodies and dilutions were used: polyclonal goat IgG anti-human SOX2 (AF2018, RnD Bio-Techne) 1:500; polyclonal rabbit IgG anti-human CDX2 (3977S, Cell Signaling Technology) 1:200; monoclonal rabbit IgG anti-human GATA3 (ab199428, Abcam) 1:200; polyclonal goat IgG anti-human E-cadherin (AF748, RnD Bio-Techne) 1:100; monoclonal mouse IgG2b anti-human POU5F1 (sc-5279, Santa Cruz) 1:100. After three washes in PBS, the secondary antibodies and DAPI were added for 1 hour at room temperature. Phalloidin conjugated with iFluor647 (Cat#ab176759, Abcam) was optionally added during the incubation with secondary antibodies. The samples were washed in PBS, then imaging was performed in PBS without mounting.

#### Imaging

Brightfield and wide-field fluorescence images, plate scanning and time-lapse imaging was performed using Nikon Eclipse Ti-E system. The individual tiles after plate scanning were stitched followed by normalisation of contrast and intensity. Confocal fluorescence imaging was done using Nikon A1-R microscope. Acquisition and processing of images was done using Nikon Elements and Fiji ImageJ2 software.

#### Bulk RNA sequencing

For bulk RNAseq, total RNA was purified using RNeasy kit (Qiagen) followed by treatment with Turbo DNase (Thermo Fisher Scientific). 500ng-1μg RNA was reverse transcribed with SuperScript II system and oligo-dT primer (Thermo Fisher Scientific), followed by 8 cycles of amplification using KAPA HiFi HotStart ReadyMix PCR Kit (KAPA Biosystems, Roche). 100-200pg of DNA were used for tagmentation performed with Nextera XT kit (Illumina), followed by 10 cycles amplification whereby iNext sequencing adaptors were added. The libraries were purified using AMPure XP magnetic beads (Beckman Coulter). The quality of the libraries was tested using Agilent Bioanalyzer system.

Bulk RNAseq was sequenced as 2x50bp paired-end reads (Illumina NovaSeq6000) and processed as follows. Raw reads were pre-processed with Trim Galore (v0.6.5) to remove Nextera adapter sequence and poor quality basecalls. Trimmed reads were aligned to the human GRCh38 genome using HISAT2 with the following options: “--no-softclip --no-mixed --no-discordant”, using gene models from Ensembl release 87. BAM files were then imported into SeqMonk (v1.46.0) data analyser to generate per-gene raw reads expression matrix and for the initial data exploration.

Bioinformatic analysis was done using RStudio software (R CoreTeam, 2020). ggplot2 package was used for data visualisation (Wickham, 2016). Differential gene expression analysis was done using DESeq2 package (Love et al., 2014) with the significance cut-off FDR<0.05. K-means clustering algorithm was applied for unsupervised clustering using pheatmap package (Kolde, 2019). Gene ontology analysis was performed using Enrichr web tool (Xie et al., 2021).

Integration of our data with published RNAseq datasets GEO:GSE166401 (Guo et al., 2021) and GEO: GSE144994 (Io et al., 2021) was done by cross-normalisation using DESeq2 (Love et al., 2014) considering all protein-coding genes. Principle component analysis was done using Factoextra 1.0.7 package (Kassambara and Mundt, 2020) considering 1000 most variable genes.

#### Single-cell 10X sequencing

For 10X single-cell RNAseq, the cells were dissociated by incubating with 0.25% trypsin for 10min at 37°C, and then an equal volume of 0.1mg/ml collagenase IV (ThermoFisher Scientific) was added for further 20min incubation at 37°C. During this incubation, the cells were triturated by pipetting with 200μl tip every 10min. The cells were resuspended in DMEM/F12 supplemented with 0.1% BSA, washed twice and then filtered through 30μm mesh. 16,000 cells were resuspended in 47μl DMEM/F12 supplemented with 0.04% BSA for further processing.

Single-cell RNA-seq libraries were prepared in the Cancer Research UK Cambridge Institute Genomics Core Facility using the following: Chromium Single Cell 3′ Library & Gel Bead Kit v3 (10x Genomics, PN-1000075), Chromium Chip B Kit (10x Genomics, PN-1000073) and Chromium Single Cell 3' Reagent Kits v3 User Guide (Manual Part CG000183 Rev C, 10X Genomics). Cell suspensions were loaded on the Chromium instrument with the expectation of collecting gel-beads emulsions containing single cells. RNA from the barcoded cells for each sample was subsequently reverse-transcribed in a C1000 Touch Thermal cycler (Bio-Rad) and all subsequent steps to generate single-cell libraries were performed according to the manufacturer’s protocol with no modifications. cDNA quality and quantity were assessed with Agilent TapeStation 4200 (High Sensitivity 5000 ScreenTape) after which 25% of material was used for gene expression library preparation.

Library quality was confirmed with Agilent TapeStation 4200 (High Sensitivity D1000 ScreenTape to evaluate library sizes) and Qubit 4.0 Flourometer (ThermoFisher Qubit™ dsDNA HS Assay Kit to evaluate dsDNA quantity). Each sample was normalized and pooled in equal molar concentration. To confirm concentration pool was qPCRed using KAPA Library Quantification Kit on QuantStudio 6 Flex before sequencing. Pool was sequenced on S2 flowcell on Illumina NovaSeq6000 sequencer with following parameters: 28 bp, read 1; 8 bp, i7 index; and 91 bp, read 2.

Single-cell 10X RNA-seq samples were processed using the CellRanger count pipeline (v3.1.0) as Single Cell 3’ (v3) data using default parameters. The resulting data were analysed using Seurat package V4.0.1 (Hao et al., 2021). The cells were filtered using the following quality control thresholds: proportion of the largest gene not more than 5%; proportion of mitochondrial transcripts not more than 10%; number of detected features between 2,500 and 6,500 (AP3 sample) or between 2,000 and 6,000 (AP8 sample); the thresholds were chosen based on the distribution across the cell population. The data were normalised and scaled, followed by the principal component analysis. Unsupervised clustering was done using the first 14 (AP3 sample) or 10 (AP8 sample) principal components, k-parameter 30, resolution 0.1 (AP3 sample) or 0.05 (AP8 sample). The markers were identified using FindMarkers command with ROC test option. Individual datasets were combined using “merge” function in Seurat followed by log-normalisation. Semi-supervised clustering analysis was done using SCINA package (Zhang et al., 2019).

#### Integration of embryo-derived single-cell RNA sequencing datasets

Embryo-derived RNAseq datasets were processed using Seurat package V4.0.1 (Hao et al., 2021). Unprocessed single-cell RNAseq dataset from *in vitro* cultured human embryos (Xiang et al., 2020) was obtained from GSE136447, only protein-coding genes were considered for the analysis. The cells were filtered for the number of detected genes (not less than 4,500 genes per cell expressed at log_2_FPKM > 2; the threshold was chosen based on the overall distribution of the number of detected genes across the cells); and for the proportion of the largest gene (< 5%). 523 out of 555 cells passed the quality control filters. Then, the dataset was first divided into two major groups of cells using unsupervised clustering corresponding to (1) inner cell mass (hsICM) and trophectoderm (hsTE), and (2) primitive endoderm (hsPrE) and epiblast (hsEPI) lineages. Then classification of each group of cells was refined further using unsupervised clustering approach. The following cell types were assigned within hsICM-hsTE lineage: hsICM, hsTE, hsCTB (cytotrophoblast), hsEarly-STB (early syncytiotrophoblast), hsSTB, hsEarly-EVT (early extravillous trophoblast), hsEVT. The following cell types were assigned within hsPrE-hsEPI lineage: hsPrE, hsPrE/PreEPI (intermediates combining properties of hsPrE and hsEPI), hsPreEPI (preimplantation epiblast), hsPostEPI-E1 and -E2 (two clusters of post-implantation epiblast), hsPostEPI-Gast (primitive streak anlage cells), hsPostEPI-AME (intermediates between epiblast and amnion cells), hsAME-E (early amniotic epithelium). Intermediates such as hsEarly-STB, hsEarly-EVT, hsPrE/PreEPI were not considered in the following analysis for clarity.

Single-cell RNA sequencing of cynomolgus macaque gastrulating embryos (Ma et al., 2019) was downloaded from GSE130114 as a processed raw counts expression matrix. The cells were filtered for the number of detected genes (not less than 3,000 genes expressed at log_2_FPKM > 1; the threshold was chosen based on the overall distribution of the number of detected genes across the cells); and for the proportion of the largest gene (< 5%). 1,229 out of 1,453 cells passed the quality control filters. Unsupervised clustering identified cells of trophectoderm and epiblast lineages, then each of them was classified using unsupervised approach to more refined cell categories. The following cell types were identified within trophectoderm lineage: cyCTB, cyEVT, cySTB. The following cell types were identified within epiblast lineage: cyPostEPI-L1, -L2, -L3, -L4. The rest of the cells were annotated according to the original publication including primitive endoderm (cyPrE), gastrulating cells (cyGast1, -2, -3) and three subpopulations of the amnion lineage (cyAME-L1, -L2, -L3). Extraembryonic mesenchyme cells (EXMC) were not considered for further analysis for clarity.

Single-cell RNAseq data from human *in utero* gastrulating embryo were kindly provided by Antonio Scialdone and Shankar Srinivas (Tyser et al., 2021). The cells annotated as epiblast, primitive streak and ectoderm were selected for the analysis. The cells were filtered for the number of detected genes (not less than 3,000 genes expressed at log_2_FPKM > 1; the threshold was chosen based on the overall distribution of the number of detected genes across the cells); and for the proportion of the largest gene (< 5%). 291 of 359 cells passed the quality control filters. Unsupervised clustering revealed a group of five primordial germ cells, which were excluded from the analysis. The remaining cells were classified using unsupervised approach to: hsPostEPI-L5 (post-implantation epiblast), hsGast5 (gastrulating cells), hsNNEC1 and hsNNEC2 (2 clusters of non-neural ectoderm). We noticed that our hsNNEC1/2 clusters corresponded to non-neural ectoderm and amniotic ectoderm, respectively, in the original publication (Tyser et al., 2021). Our diffusion plot analysis revealed a single trajectory within this cell population (Figure 1) and therefore we considered these cells as a single lineage.

The quality control-filtered and annotated single-cell RNAseq datasets were combined using “merge” function in Seurat package. One-to-one orthology was used to combine human and cynomolgus monkey data. 1,461 cells assigned to 30 cell types were present in the integrated embryo dataset.

To compare the *in vitro* cultured cells to the embryo, pseudobulk gene expression of the *in vitro* cell clusters identified in 10X RNAseq was combined with single-cell RNAseq data from embryos. Principle component analysis was done using Factoextra 1.0.7 package (Kassambara and Mundt, 2020), considering either most variable genes of the embryo dataset, or most variable genes between hPSC and AME-E-like cells obtained from bulk RNAseq analysis. Fractions of identity were calculated using DeconRNASeq package (Gong and Szustakowski, 2013) considering all expressed protein-coding genes. Diffusion map analysis was performed using Destiny package (Angerer et al., 2016), considering 1,000 most variable protein-coding genes.

### Quantification and statistical analysis

Bulk RNA sequencing was performed in two biological replicates whereby AME-E-like cells differentiation was induced using HNES1 cell line in two independent rounds. Differential expression analysis was done using the cut-off of FDR <0.05.

10X single-cell RNA sequencing was done using one sample generated from HNES1 cell line per condition, aiming to sequence 10,000 cells per sample. Markers of subpopulations were identified using ROC analysis using default parameters in FindMarkers Seurat function: logfc.threshold = 0.25, min.pct = 0.1.

qRT-PCR results are shown for two biological replicates per condition in Figures 2C, 3, and 4 biological replicates per condition in Figure 4D, otherwise 2 technical replicates.

Clonogenicity assay in Figure S3D was done in two biological replicates.

Statistical significance of Gene Ontology terms in Figures 3C and 5D was calculated using Benjamini-Hochberg procedure.

Flow cytometry results in Figure 4E was reproduced in 2-3 independent rounds of differentiation.

## Data Availability

RNA sequencing data have been deposited in Gene Expression Omnibus database and are publicly available as of the date of publication. Accession numbers are listed in the key resources table. This paper analyses existing, publicly available data. The accession numbers for the datasets are listed in the key resources table. This paper does not report original code. Any additional information required to reanalyze the data reported in this paper is available from the lead contact upon request.

## Supplemental information

Document S1. Figures S1–S5

Data S1. Integrated single-cell RNA-seq of primates embryos, related to Figures 1 and 6

Data S2. Expression matrix of the integrated single-cell RNA-seq of primates embryos, related to Figure 1

Data S3. Bulk RNA-seq of partially primed hPSC induced to AME-E-like cells, related to Figure 3

Data S4. Markers of trophectoderm, early and late amnion in primate embryos, related to Figure 4

Data S5. Markers of AME-E and AME-L subpopulations (10× sequencing)

Document S2. Article plus supplemental information
