# Supplementary material for: Amniogenesis occurs in two independent waves in primates
Source: Cell Stem Cell. 2022 May 5;29(5):744–759.e6. doi: 10.1016/j.stem.2022.03.014 (PMC9627701; doi:10.1016/j.stem.2022.03.014)
Supplement: Document S1. Figures S1–S5 [file mmc1.pdf]

Cell Stem Cell, Volume *29*

Supplemental Information

Amniogenesis occurs in two independent waves in primates

Maria Rostovskaya, Simon Andrews, Wolf Reik, and Peter J. Rugg-Gunn

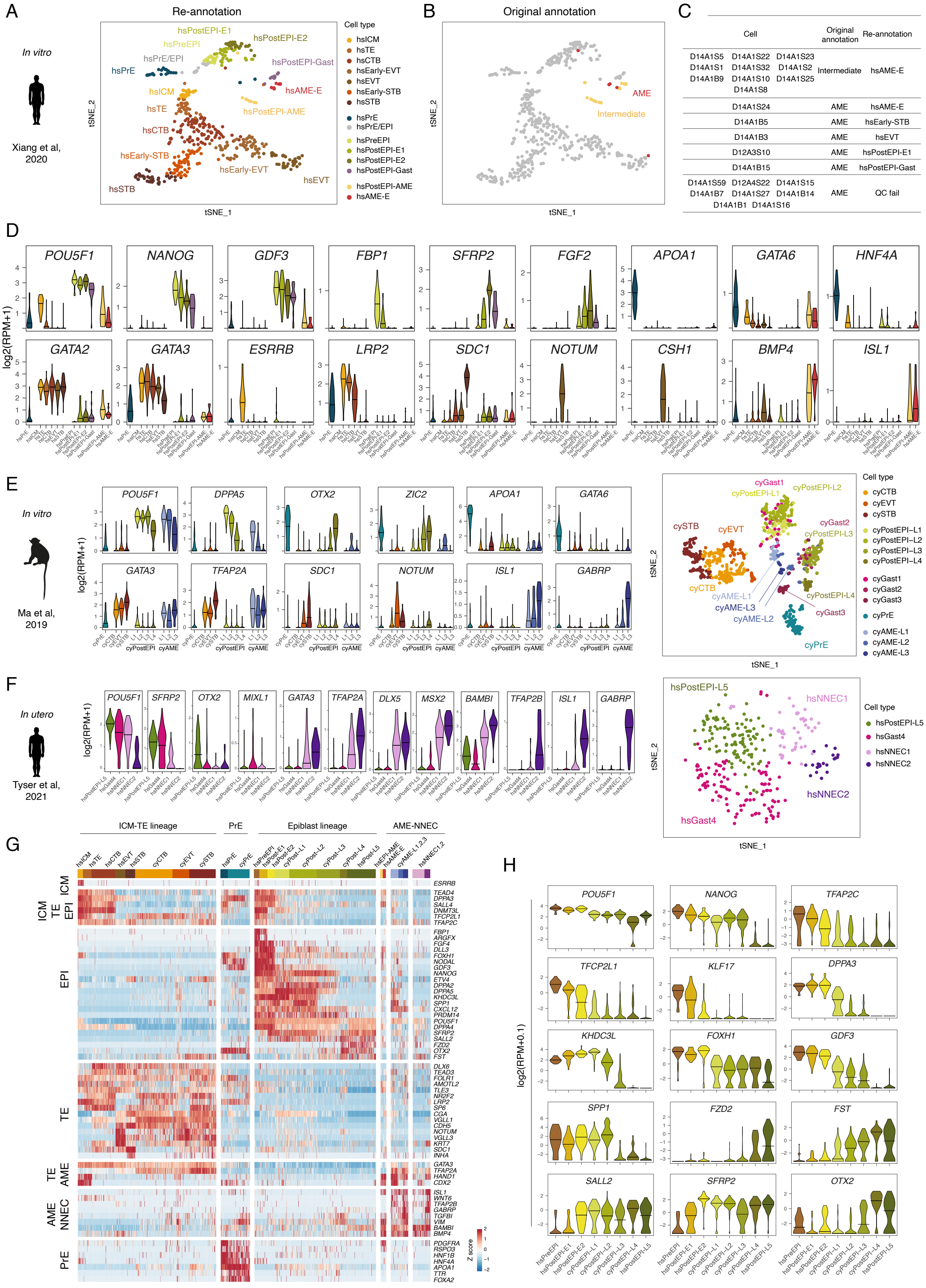

Cell			Original annotation	Re-annotation
D14A1S5 D14A1S1 D14A1B9	D14A1S22 D14A1S32 D14A1S10 D14A1S8	D14A1S23 D14A1S2 D14A1S25	Intermediate	hsAME-E
D14A1S24			AME	hsAME-E
D14A1B5			AME	hsEarly-STB
D14A1B3			AME	hsEVT
D12A3S10			AME	hsPostEPI-E1
D14A1B15			AME	hsPostEPI-Gast
D14A1S59 D14A1B7 D14A1B1	D12A4S22 D14A1S27 D14A1S16	D14A1S15 D14A1B14	AME	QC fail

Figure S1 (related to Figure 1). Annotation of the published single-cell RNAseq datasets obtained from human and cynomolgus monkey embryos.

(A) tSNE showing re-annotated single-cell RNAseq dataset from *in vitro* cultured human embryos (Xiang et al., 2020).
(B) tSNE showing the cells originally annotated as amniotic lineage in Xiang et al., 2020.
(C) Comparison of the original and the updated annotation of amnion cells from Xiang et al., 2020, dataset.
(D) Violin plots showing expression of markers in the re-annotated human embryos dataset.
(E) Refined annotation of single-cell RNAseq dataset from *in vitro* cultured cynomolgus monkey embryos (Ma et al., 2019): violin plots showing expression of markers and tSNE.
(F) Refined annotation of single-cell RNAseq dataset from *in utero* gastrulating human embryo (Tyser et al., 2021): violin plots showing expression of markers and tSNE.
(G) Heatmap showing selected markers in the integrated RNAseq dataset of primate embryos.
(H) Violin plots showing expression of markers during the epiblast progression across datasets.

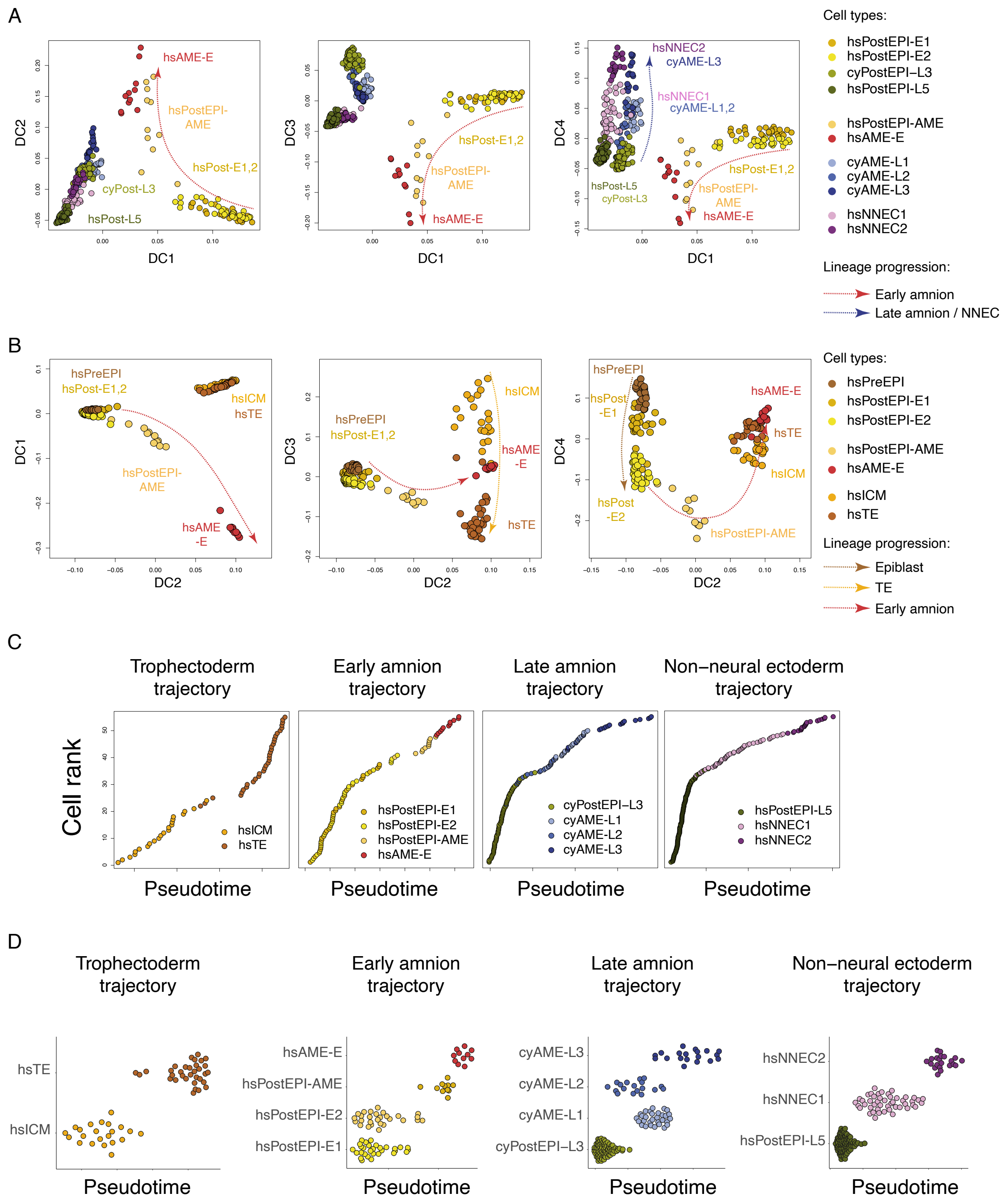

Figure S2 (related to Figure 1). Developmental trajectories in primates embryos.

(A) and (B) Diffusion maps of the selected lineages from the integrated single-cell RNAseq of primates embryos.

(C) Individual trajectories of trophectoderm (DC3), early amnion (DC1), late amnion (DC4) and non-neural ectoderm (DC4) lineages.

(D) Transitions of cell types in pseudotime.

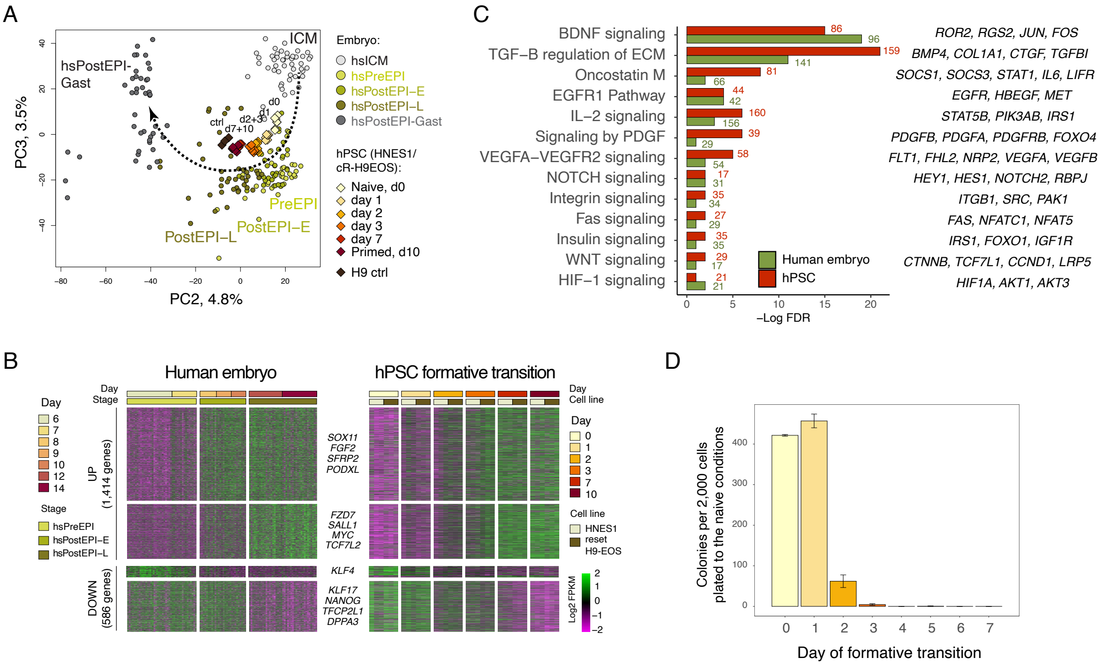

Figure S3 (related to Figure 2). The *in vitro* formative transition recapitulates epiblast progression in human embryos

(A) PCA of epiblast lineage in human embryos and hPSC *in vitro* during the formative transition. Note that PC1 distinguishes individual datasets and therefore not shown.

(B) Heatmap showing expression of 2,000 most variable genes during the epiblast progression in human embryo, in human epiblast and in hPSC during the *in vitro* formative transition.

(C) Gene ontology terms related to signalling pathways enriched in genes upregulated in epiblast and in hPSC during naive-to-primed transition.

(D) Clonogenicity assay to test the abilities to self-renew of hPSC during the formative transition.

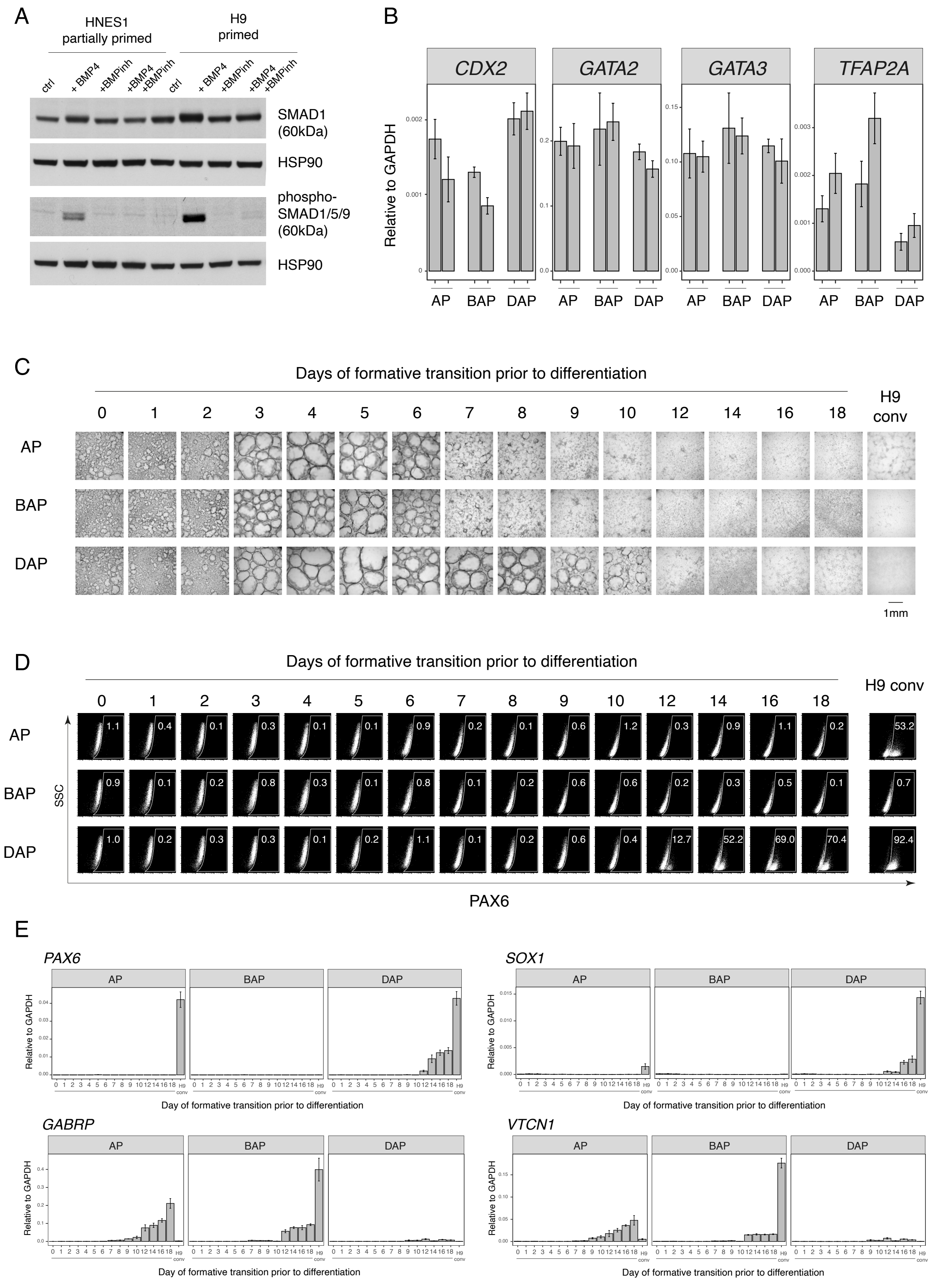

Figure S4 (related to Figure 2). hPSC change the response to BMP4 signalling during the formative transition

(A) Western blot for total SMAD1, phosphorylated SMAD1/5/9 and a house-keeping gene HSP90 in partially primed or primed hPSC in the indicated conditions.
(B) qRT-PCR for markers in partially primed hPSC differentiated in AP, in AP combined with BMP4 (“BAP”), or AP with BMP inhibitor DM3189 (“DAP”).
(C) Bright field images, (D) flow cytometry for PAX6 and (E) qRT-PCR for markers in hPSC after different periods of the formative transition and conventional H9 hESC differentiated in AP, BAP or DAP.
Note: the panel of brightfield images in Figure S4C in AP condition is the same as in Figure 2I.

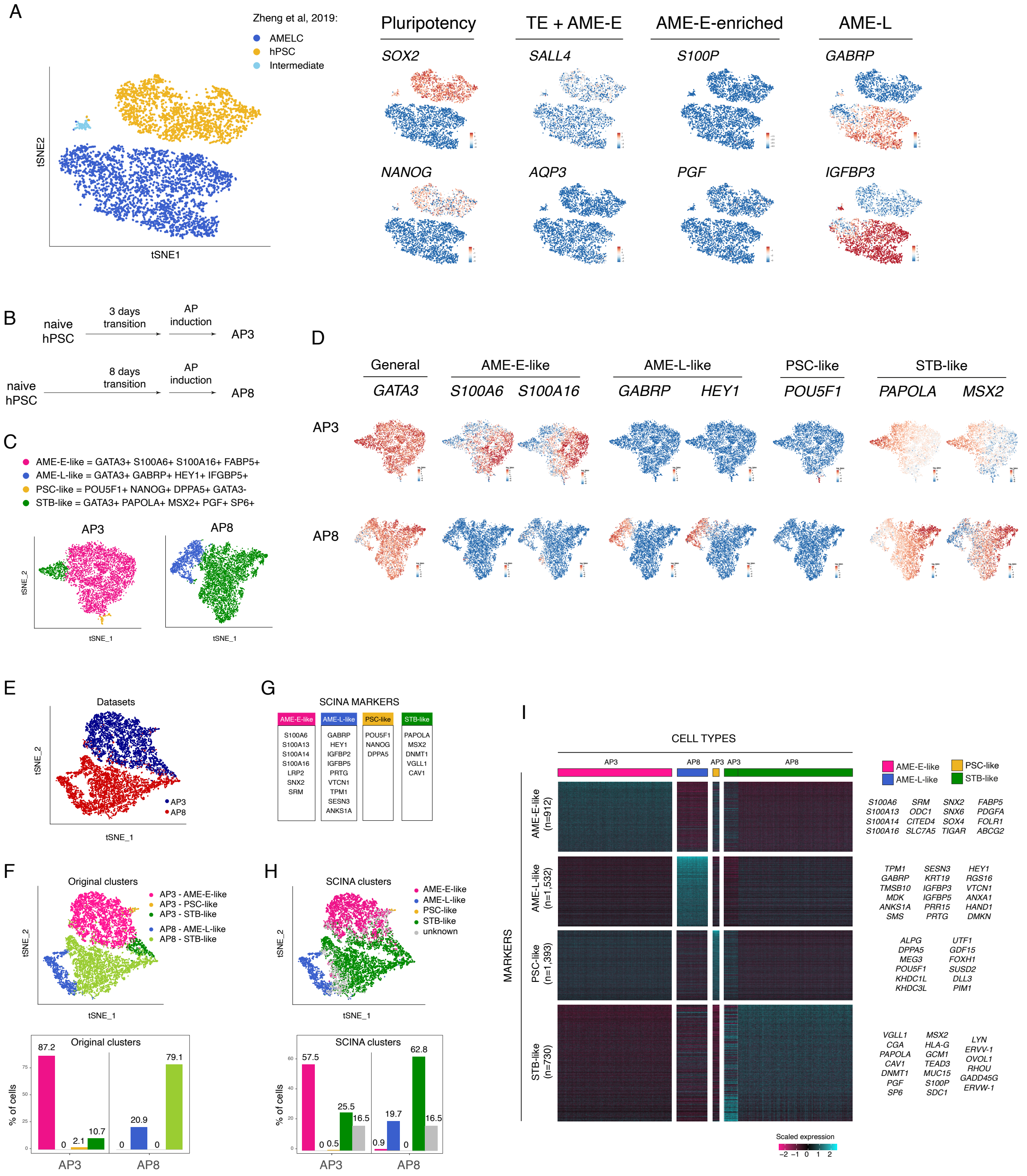

Figure S5 (related to Figure 5). Characterisation of AME-E-like and AME-L-like cells obtained by *in vitro* differentiation from partially primed and primed hPSC at the single cell level

(A) Expression of markers in amnion-like cells derived by BMP4 treatment of hPSC (Zheng et al, 2019).

(B) Experimental setup.

(C) Cell categories identified in AP-treated partially primed and primed hPSC.

(D) Expression of selected markers in AP-treated partially primed and primed hPSC.

(E) tSNE of the dataset combining AP-treated partially primed and primed hPSC.

(F) tSNE showing the original cell categories identified in each individual dataset; bar plot shows their frequencies.
(G) List of markers of the identified categories of cells using for SCINA analysis.
(H) SCINA analysis. Bar plot shows frequencies of the identified cell categories in the population.
(I) Heatmap showing markers of the cell categories identified by unsupervised clustering in the combined dataset.
